# Extreme Arsenic Bioaccumulation Factor Variability in Lake Titicaca, Bolivia

**DOI:** 10.1038/s41598-019-47183-8

**Published:** 2019-07-23

**Authors:** Géraldine Sarret, Stéphane Guédron, Dario Acha, Sarah Bureau, Florent Arnaud-Godet, Delphine Tisserand, Marisol Goni-Urriza, Claire Gassie, Céline Duwig, Olivier Proux, Anne-Marie Aucour

**Affiliations:** 1 ISTerre (Institut des Sciences de la Terre), Univ. Grenoble Alpes, CNRS, IRD, IFFSTAR, Univ. Savoie Mont Blanc, 38000 Grenoble, France; 2 Instituto de Ecología, Unidad de Calidad Ambiental (UCA), Carrera de Biología, Universidad Mayor de San Andrés, Campus Universitario de Cota Cota, casilla La Paz, 10077 Bolivia; 3 Université Lyon 1, ENS de Lyon, CNRS, UMR 5276 LGL-TPE, F-69622 Villeurbanne, France; 4 Environmental Microbiology, CNRS/ UNIV PAU & PAYS ADOUR/E2S UPPA, Institut des sciences analytiques et de physicochimie pour l’environnement et les matériaux, IPREM, UMR5254 Pau, France; 5 Univ. Grenoble Alpes, CNRS, IRD, IGE, Grenoble, F-38 000 France; 6 OSUG (Observatoire des Sciences de l’Univers de Grenoble), Univ. Grenoble Alpes, CNRS, IRD, 38041 Grenoble, France

**Keywords:** Environmental microbiology, Element cycles, Natural hazards

## Abstract

Latin America, like other areas in the world, is faced with the problem of high arsenic (As) background in surface and groundwater, with impacts on human health. We studied As biogeochemical cycling by periphyton in Lake Titicaca and the mine-impacted Lake Uru Uru. As concentration was measured in water, sediment, totora plants (*Schoenoplectus californicus*) and periphyton growing on stems, and As speciation was determined by X-ray absorption spectroscopy in bulk and EDTA-extracted periphyton. Dissolved arsenic was between 5.0 and 15 μg L^−1^ in Lake Titicaca and reached 78.5 μg L^−1^ in Lake Uru Uru. As accumulation in periphyton was highly variable. We report the highest As bioaccumulation factors ever measured (BAFs_periphyton_ up to 245,000) in one zone of Lake Titicaca, with As present as As(V) and monomethyl-As (MMA(V)). Non-accumulating periphyton found in the other sites presented BAFs_periphyton_ between 1281 and 11,962, with As present as As(III), As(V) and arsenosugars. DNA analysis evidenced several taxa possibly related to this phenomenon. Further screening of bacterial and algal isolates would be necessary to identify the organism(s) responsible for As hyperaccumulation. Impacts on the ecosystem and human health appear limited, but such organisms or consortia would be of great interest for the treatment of As contaminated water.

## Introduction

The arsenic (As) geogenic background of surface and ground-water is naturally high in South America, predominantly originating from young volcanic rocks and their weathering products in arid oxidizing conditions^1–4^. As a result, about 4.5 million people in South America are chronically exposed to high levels of As (>50 µg L^−1^)^5^, and certain Andean populations have developed a unique capacity to adapt to As toxicity^6,7^. Concerning Andean lakes, extreme As concentrations are observed in hypersaline lakes colonized by extremophile bacterial communities^8^, and lower but still significant concentrations are observed in other, less saline lakes, which are major freshwater resources^5^. In many areas of the Altiplano, mining and smelting activities add to natural rock weathering processes in the As budget^9^.

The biogeochemical cycling of As has been studied in freshwater and marine ecosystems, and in hypersaline environments, but its trophic transfer and speciation in living organisms mainly concerns the marine environment and As contaminated freshwater systems^10^. In the high altitude lakes of the Andean Altiplano (above 3500 m asl), shallow zones (<2 m) are colonized by totoras (*Schoenoplectus californicus*, syn *Scirpus californicus*). These macrophytes were used for construction purposes in traditional Andean culture. Nowadays, they are mainly used as cattle fodder and have been tested successfully in constructed wetlands in North America for the removal of metals (Zn, Cu, Cd, Pb) and nutrients from wastewater^11–14^. The filtration potential of wetland plants does not rely on absorption by the plant, but on physico-chemical and biologically driven processes taking place on submerged stems and in the rhizosphere^15,16^. In particular, the periphyton, an assemblage of algae and bacteria forming a biofilm on submerged plant shoots, is known to play a key role in contaminant removal in constructed wetlands^17^. The periphyton was shown to act as a sink for methylmercury in Lake Titicaca^18^ and Lake Uru Uru^19^ (Bolivia). Recently, consortia of algae and bacteria specifically designed for wastewater treatment have been proposed^20,21^. Furthermore, periphyton is at the base of trophic chains in freshwater ecosystems, and plays a key role in nutrient cycling^22^. Better understanding of the interactions between contaminants and organisms forming these assemblages could help to improve the efficiency of water treatment and evaluate potential trophic transfer issues in natural systems.

The purpose of this work was to decipher the role of periphyton in the biogeochemical cycle of As in two lakes of the Andean Altiplano with contrasting As levels: (i) Lake Titicaca, which has moderate dissolved As level (about 10 µg L^−1^); and (ii) Lake Uru Uru, which has elevated concentrations (about 80 µg L^−1^) due to a higher geogenic background and to anthropogenic discharges (i.e., mining and smelting activities). After determining the physico-chemical parameters of the water and elemental contents in each compartment (water, sediment, totora plants and periphyton growing on totora shoots), we focused on the speciation and localization of As in periphyton using X-ray absorption spectroscopy coupled with EDTA extractions to separate the intra and extracellular pools of As. In parallel, the diversity of the periphyton was studied by 16 S rDNA sequencing. A discussion is provided on the abiotic vs. biotic origin of As accumulation, as well as the (eco)toxicological implications, and the possible applications of this periphyton in phytotechnologies for water treatment.

## Results

### Arsenic in lake water and sediments

**Figure 1:**
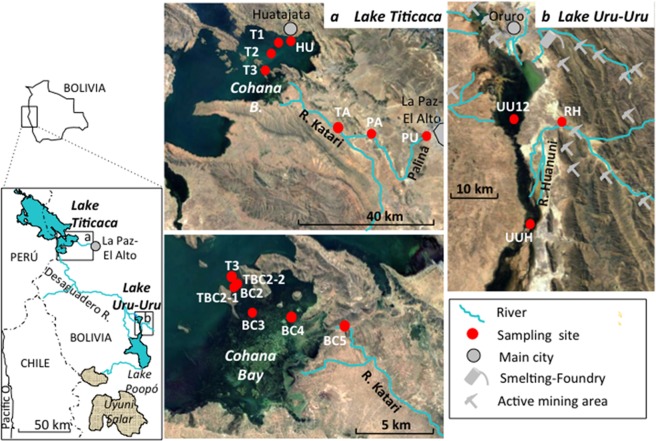
Map of the area of Lake Titicaca and Uru Uru in the Bolivian Altiplano, with sampling sites. Map data: Google, DigitalGlobe.

In Lake Titicaca, the sampling was performed along a transect from the outlet of the Katari River, across Cohana Bay, a shallow area (0.5 to 2 m depth) of about 35 km2 with a rather eutrophic character, colonized by totoras, then across the open part of the lake up to Huatajata in the other side, also colonized by totoras (Fig. 1, see also Methods and Supplemental Table S1).

**Figure 2:**
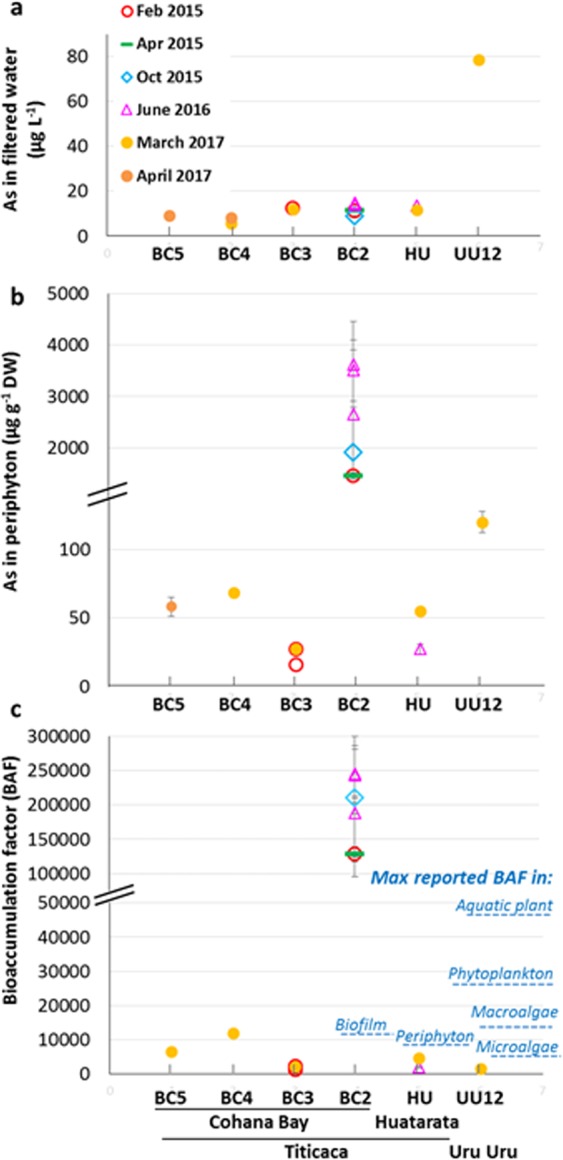
Total As concentration in filtered water (**a**) and in periphyton (**b**). (**c**) As bioaccumulation factor (BAF_periphyton_) in the periphyton (data in Supplemental Table S5), and comparison with previously reported values (values given in Supplemental Table S6). Points TBC2-1 and TBC2-2 are included in the BC2 point.

**Figure 3:**
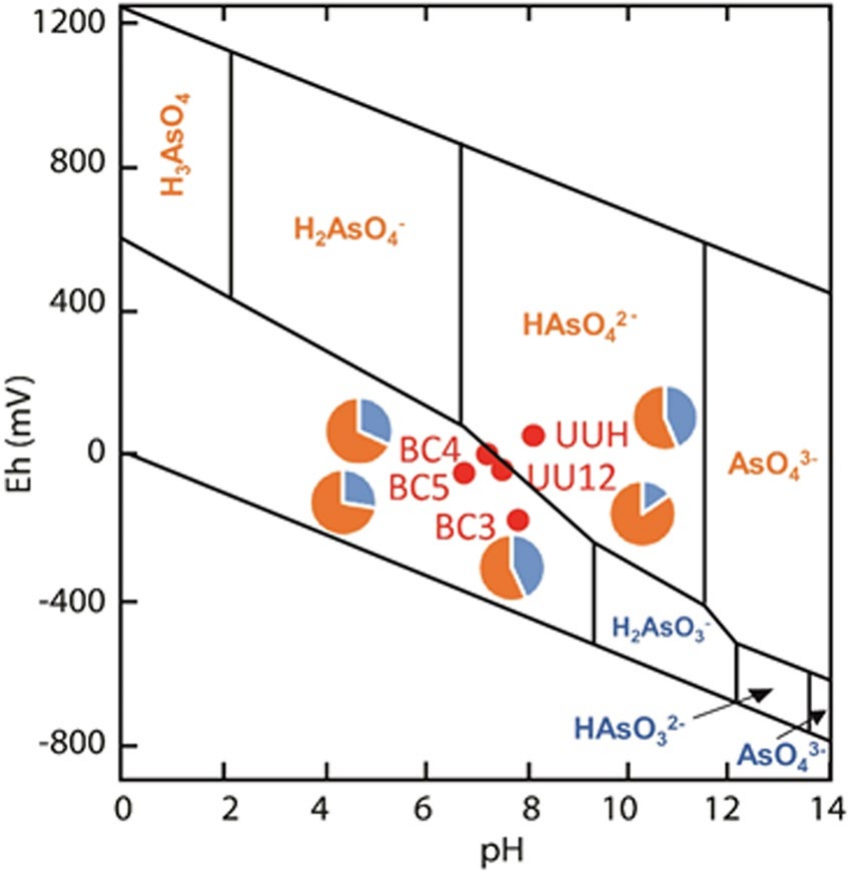
Eh-pH diagram for As, and data for the water samples collected during the PB5 campaign. The proportions of As(III) and As(V) for each sample appear as a pie chart beside the sample name, with As(III) species in blue and As(V) species in orange.

The As concentration in filtered lake waters together with other key chemical parameters (Fe, Mn, pH, E_h_, dissolved oxygen, salinity, Cl^−^, S, SO_4_^2−^, dissolved hydrogen sulfides (ΣH_2_S), NO_3_^−^, PO_4_^3−^, Na, Ca and dissolved organic carbon) are given in Supplemental Table S2. The lake water is slightly alkaline (pH 6.7 to 8.5) and saline (0.3 to 0.8 g kg^−1^). The sulfur content is relatively high and is mostly present as sulfate (131 to 277 mg L^−1^ with a mean of 226 ± 59 mg L^−1^) and a minor fraction of dissolved H_2_S. Conditions were in general oxic to sub-oxic in the first meter below the surface, but more reduced during an algal bloom in April 2015 (PB2). The level of As in filtered water was close to or slightly above 10 µg L^−1^ (Fig. 2, Supplemental Table S2), defined as the World Health Organization (WHO) threshold limit for drinking water. This As level can be considered normal compared to freshwater systems used for drinking water in Latin America. The levels of other dissolved metals were generally low to very low except for Fe and Mn during a rainfall event (PB5). Similar As concentrations were found in the Katari River and its main tributary, the Palina River, with a slight downstream decrease in dissolved As from the Katari River to Cohana Bay (11.0, 9.0 and 8.2 µg L^−1^ at PA, BC5 and BC4, respectively, in April 2017). The rainwater contained a very low level of As (<0.2 µg L^−1^). Slight variations in filtered As were measured over time (i.e., between 8.0 and 14.9 µg L^−1^ at BC2), with no evident relationship with the lake level and between sampling sites (i.e., from 5.7 µg L^−1^ at BC4 to 12.1 µg L^−1^ at BC3 in the March 2017 campaign). The proportion of As(V) and As(III) in filtered water was determined with an As speciation cartridge (MetalSoft) retaining As(V). The results showed that As(V) dominates As(III), both in the lake (measurements done at BC3 and BC4) and the Katari River (BC5) (i.e., As(V) between 60 and 70%, Fig. 3). This distribution disagrees with the expected predominance of As(III) given by the Pourbaix diagram for the pH and E_h_ conditions measured (Fig. 3). This discrepancy might be explained by kinetic factors: (1) the equilibration of As(III)/As(V) is slow in case of change in E_h_^23^ [and refs therein], and (2) redox conditions fluctuate daily in the water column, with the daytime photosynthetic activity of phototrophs counterbalanced by oxygen consumption via respiration and H_2_S oxidation^19,24^. In addition, the complexation of As(V) by organic matter and iron^25,26^ are not considered in this Pourbaix diagram.

The sediments collected in Cohana Bay presented an organo-detrital facies in the bay (e.g., BC3) and a carbonate rich facies at the boundary with open waters (i.e., BC2) due to the higher abundance of *Characeae*^27,28^. As content was rather low (21 to 62 µg g^−1^, up to 60 µg kg^−1^ - Supplemental Table S3) and in the range of suspended particles and sediments reported for the Katari River^29^. Regarding the entire Cohana Bay, acid volatile sulfide (AVS) content was generally elevated as a function of sediment depth (28 to 56 µmol g^−1^, Supplemental Table S3). Fe was the main simultaneously extracted metal. Thus, the sulfide pool of the sediment was dominated by FeS content, although organic sulfides may also be present, as previously reported in Lake Titicaca^30^.

Downstream in the endorheic basin, the upper (UU12) and lower (UUH) part of Lake Uru Uru were sampled, as was an acid mine drainage tributary (Huanuni River, RH) (Fig. 1, See also Methods and Supplemental Table S1). The As content in filtered waters and other key chemical variables are shown in Supplemental Table S2. At UU12, the dissolved As levels of this lake (78.5 ± 5.5 µg L^−1^) were 8-fold higher than in Lake Titicaca, with a slightly higher proportion of As(V) (85%) (Fig. 3). The sulfur level was 70% higher (142 ± 0.9 mg L^−1^ S at UU12), with a comparable proportion of sulfate (88% of total S at UU12). The sediment contained more AVSs (200 to 319 µmol g^−1^), which is consistent with the higher S content in water, and was mostly composed of FeS (Supplemental Table S3). The higher As and S concentrations in Lake Uru Uru at UU12 compared to Titicaca can be partly explained by a concentration effect due to a higher evaporation, since the salinity was twice as high. Furthermore, upstream mining activities releasing acid mine drainage (AMD) into the lake via the Huanuni River (RH), Tagarete River and other tributaries^9^, are likely the main source of As and S. The Huanuni River contained 84 ± 10 µg L^−1^ present as As(III) only, and 376 ± 0.6 mg L^−1^ S, and had a chemical composition typical of AMD^9^. The proportion of As(III) at UU12 was higher than predicted by the Pourbaix diagram (Fig. 3). The presence of an extremely high dissolved Fe concentration (about 111 mg L^−1^), with possibly a significant proportion of Fe(II) and colloidal Fe, and the presence of reduced sulfur species (Supplemental Table S2) is assumed to participate in maintaining As in solution and in its reduced state^23,26^. Downstream, at the outlet of the lake (UUH), dissolved As was much lower As (4.8 ± 0.4 µg L^−1^), with 44% present as As(III) (Fig. 3). This significant decrease in dissolved As content likely results from the precipitation of As with Fe oxyhydroxides, since the conditions were more oxic (E_h_ = 132 mV) and the sediment was enriched in As and Fe and depleted in AVSs (181 ± 3 µg g-1 As, 4.72 ± 0.11% Fe and 130 µmol g^−1^ AVS, respectively) compared to UU12 (76 ± 8 µg g-1 As, 2.83 ± 0.17% Fe and 217 µmol g^−1^, AVSs, Supplemental Table S3).

### Arsenic in totoras

**Figure 4:**
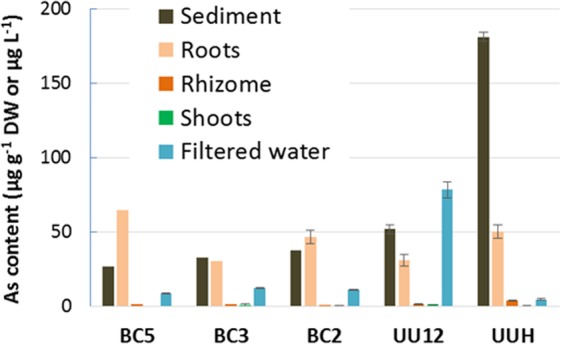
As concentrations in the bulk sediment and totora plant parts (μg g^−1^DW) and in filtered water (μg L^−1^). For the sediment, As concentrations were obtained by merging the concentrations measured in the 0–3, 3–10 and 10–20 cm layers. Raw values are given in Tables S2, S3 and S4.

Totora plants showed strong partitioning of As in roots, and very limited transfer to rhizome and shoots. As contents in roots and shoots ranged from 30.8 to 65.0 and 0.4 to 1.5 µg g^−1^ DW, respectively (Fig. 4, Supplemental Table S4). Hence, the As bioaccumulation factor (BAF_totora_ = [As]_shoot_/[As]_sediment_) was extremely low (0.002 to 0.031). The high Fe content in roots (717 to 28,081 mg kg^−1^) suggests the presence of a root plaque, which could sorb a fraction of root As, as observed for other aquatic plants^31^.

### Arsenic in periphyton

Compared to totora shoots, As content in the periphyton was generally high, ranging from 16 to 69 ± 2 µg g^−1^ DW in Lake Titicaca, and 120 ± 8 µg g^−1^ DW in Lake Uru Uru (UU12) (Fig. 2b, Supplemental Table S5). The most striking finding was the strong As enrichment in the periphyton collected at the outer fringe of Cohana Bay (BC2), where As content ranged from 1452 ± 66 to 2647 ± 1263 µg As g^−1^ DW. Calculated bioaccumulation factors (BAFs_periphyton_ = [As]_periphyton_/[As]_filtered water_) ranged from 128,118 to 238,854 at this latter point (BC2), compared to 1,281 to 11,962 for the other sites (Fig. 2c). Although the term hyperaccumulation is defined for plants, with a threshold at 1000 mg kg^−1^ DW in aerial parts^32^, we propose to use it for the periphyton based on the extremely high BAFs_periphyton_. Arsenic hyperaccumulation was also observed in sampling points distant by 150 m from point BC2 (i.e., TBC2-1 and TBC2-2). At these points, As contents and BAFs_periphyton_ were in the same range as in BC2, and even higher. It is noteworthy that the large standard deviations in elemental concentrations are due to periphyton heterogeneity rather than to analytical uncertainty. Indeed, periphyton samples were heterogeneous from the morphological viewpoint, which is reflected in their chemical composition (as shown by duplicate µXRF analyzes of freeze-dried periphyton, see Supplemental Fig. S3), and taxonomic composition (see following sections). In addition, the periphyton collected after a rainfall event (PB5 campaign) in the Katari River near the estuary flowing into Cohana Bay (BC5) and inside the bay close to the estuary (BC4), where water was brownish, was strongly enriched in Fe and Al (one order of magnitude compared to the other samples). These concentrations, as well as the Al/Fe ratio were comparable to those measured in the SPM during the same sampling (Al/Fe = 1.8 and 2.1 for the periphyton BC4 and BC5, and 1.9 for the SPM). These observations indicate the presence of detritic particles entrapped in the biological material in these particular samples.

**Figure 5:**
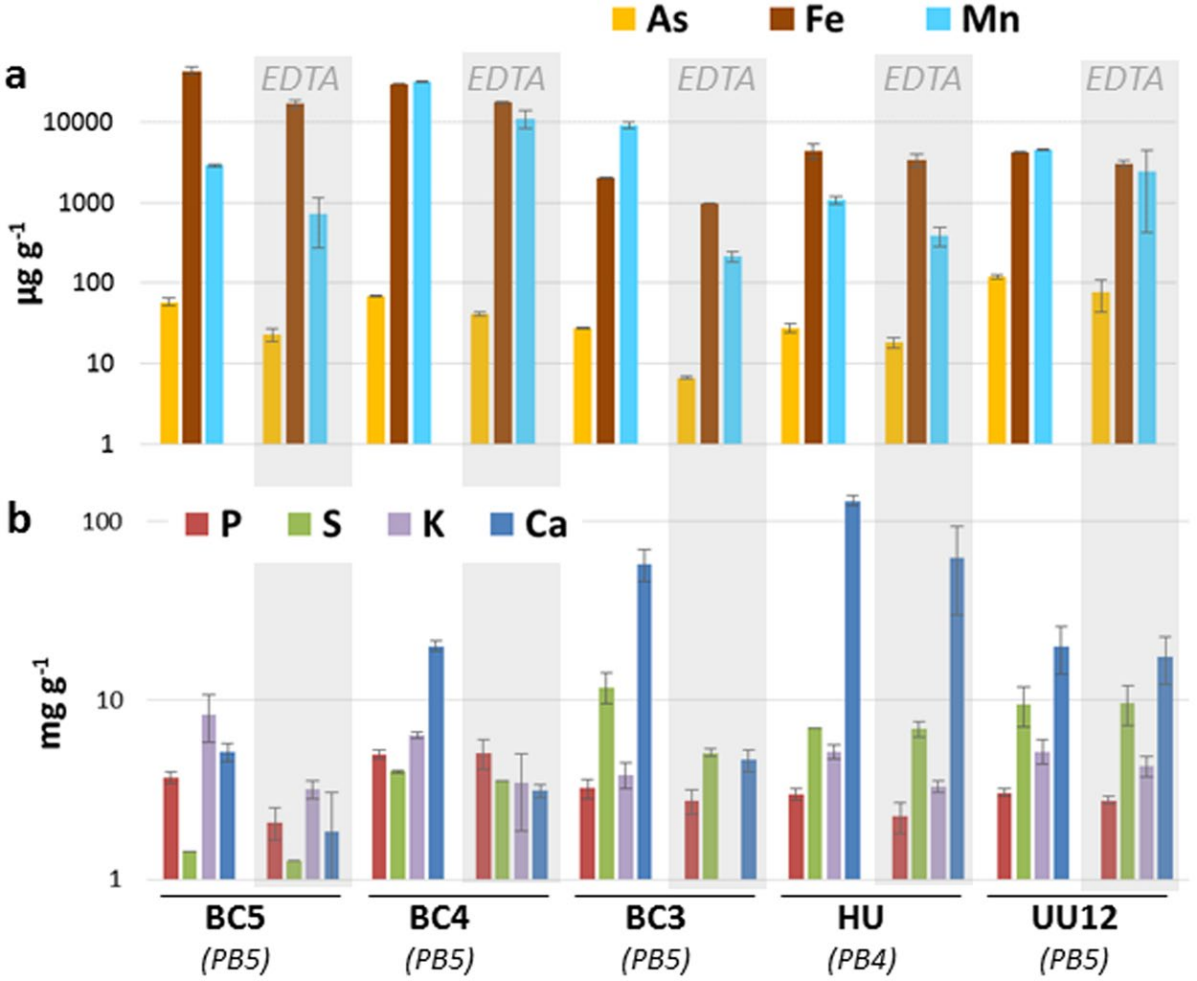
Elemental concentrations in the non hyperaccumulator periphyton samples before and after EDTA extraction. Y axis is in log scale. All concentrations are given on a dry weight basis.

Considering the entire set of periphyton samples, As enrichment was not correlated with other elements such as Fe, Mn, Ca or S. The As/Fe molar ratio was close to 1 (1.4 ± 0.8, N = 23) for the hyperaccumulating periphyton compared to 0.011 ± 0.010 for the non-accumulating periphyton (N = 16). EDTA extraction was performed to evaluate the intra- and extracellular pools of elements including As. For the non-accumulating periphyton, EDTA extracted mostly Fe, Mn and As (Fig. 5, Supplemental Table S5), which suggests the presence of As sorbed on extracellular Fe/Mn oxides. The extracellular pool of As accounted for 34 to 76% of total As depending on the samples, compared to 22 to 61% for Fe and 46 to 98% for Mn. Large quantities of Ca were also extracted by EDTA except for UU12, so As might also be associated with extracellular Ca carbonates. Unfortunately, this extraction experiment could not be performed on the hyperaccumulating periphyton because point BC2 was not accessible during the PB5 campaign.

**Figure 6:**
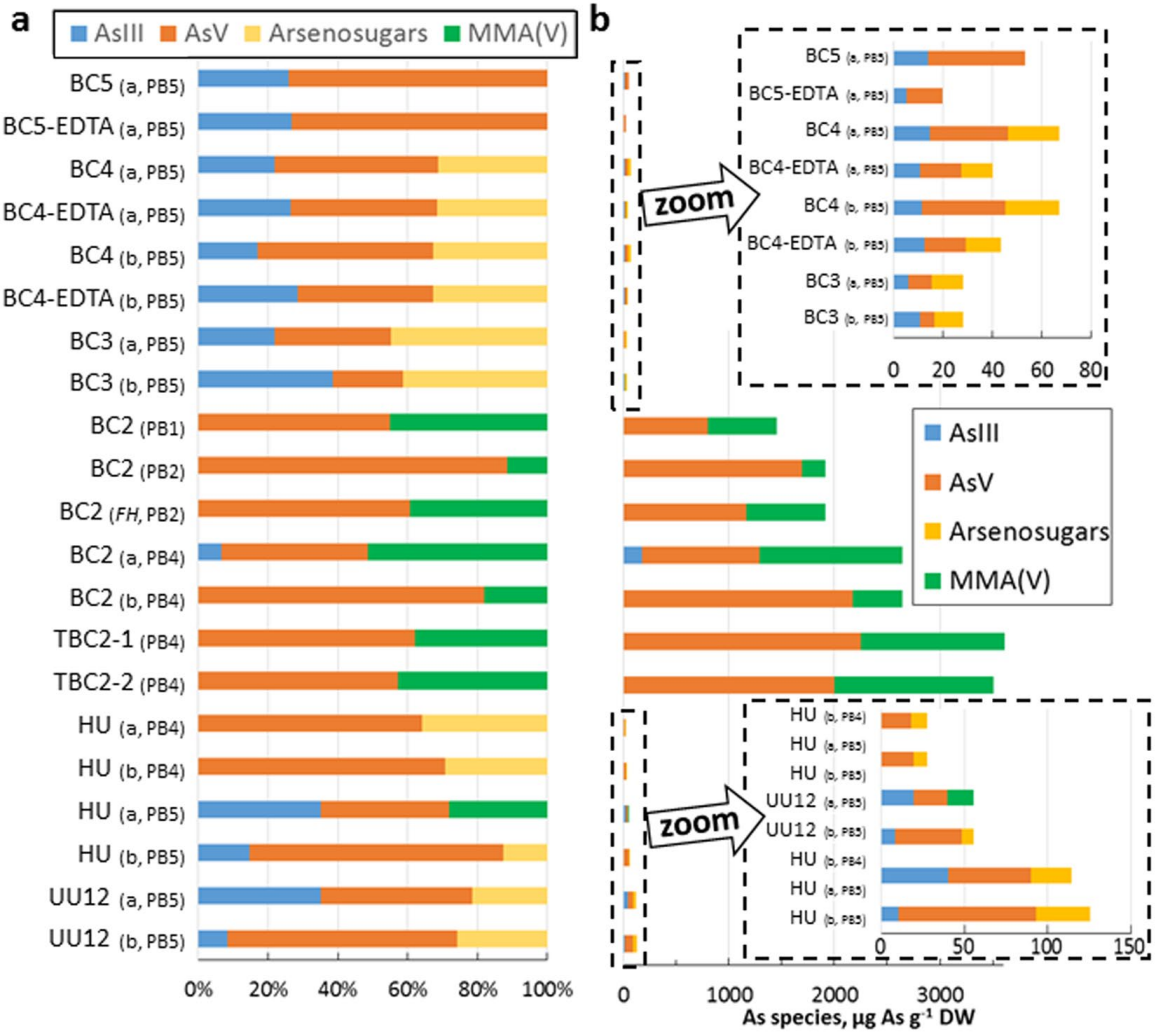
Distribution of As species in As molar percentage (**a**) and concentration (**b**) in the periphyton determined by linear combination fits of XANES spectra. EDTA: EDTA extracted periphyton, a, b: duplicate sampling and analysis of periphyton. *FH*: spectrum recorded on frozen hydrated periphyton.

X-ray absorption near edge structure (XANES) analysis of the periphyton allowed characterizing As speciation. The set of spectra was correctly reproduced by a combination of arsenite, arsenate, monomethyl As (MMA(V)) and arsenosugars (Fig. 6, Supplemental Fig. S4, Supplemental Table S7). Other As species including As(III) bound to glutathione (GSH) and thio-As species were not detected in the samples, meaning that they accounted for less than 10% of total As, which corresponds to the accuracy of the method^33^. The results obtained between replicate samples were consistent within 10% error, except for one sample (periphyton HU, BP5 campaign). The non-accumulating periphyton contained a mixture of As(III), As(V) and arsenosugars. The contribution of As(III) was visible on the XANES spectra, with significant absorbance in the 11870 eV region (Supplemental Fig. S4). Various arsenite and arsenate standards were tested in order to obtain more insight into their nature in the samples. Sodium arsenite and arsenate can be used as proxies for outer sphere (i.e., weakly bound) complexes, whereas sorbed species (As(III) and As(V)-ferrihydrite, As(V)-goethite and As(V)-calcite) correspond to inner sphere (i.e., more strongly bound) As complexes. For arsenite, best fits were obtained with either sodium arsenite or As(III)-ferrihydrite. For arsenate, sodium arsenate provided the best fits in most cases, but other As(V) references (As(V)-calcite, As(V)-ferrihydrite and As(V)-goethite) also gave satisfactory fits. Thus, in the non-accumulating periphyton, arsenite and arsenate may be present as both weakly and strongly bound complexes. For two periphyton samples (BC4 and BC5, close to outlet of the Katari River), spectra were recorded before and after EDTA extraction. The results showed that EDTA removes predominantly arsenosugars and As(V). This means that intracellular As is composed of the three As species (As(III), As(V) and arsenosugars), while extracellular As is almost entirely composed of As(V) and arsenosugars.

Based on LCFs results, the hyperaccumulating periphyton contained a mixture of As(V) and MMA(V) (Fig. 6, Supplemental Table S7). In one sample only, As(III) was present as a minor component (7%, which is within the precision of the method). The absence or very low contribution of As(III) is consistent with the shape of the As edge, with low absorbance in the 11870 eV region (Supplemental Fig. S4). Like for the non hyperaccumulating periphyton, best fits were obtained with various As(V) standards depending on the samples, so it was not possible to conclude on the exact nature of the As(V) species.

### Diversity of microbial communities in As hyperaccumulating vs non-accumulating periphyton

**Figure 7:**
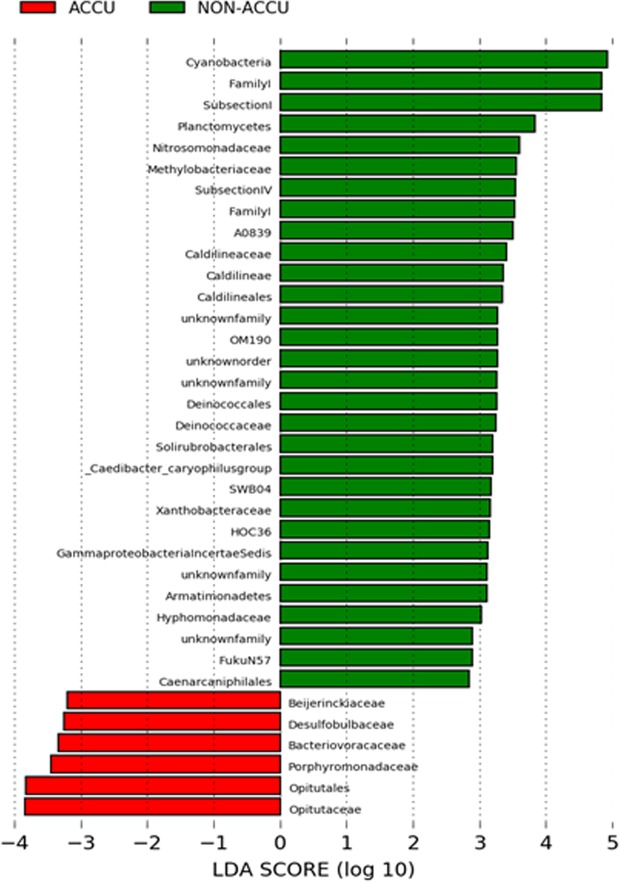
Lefse analysis of taxa differentially abundant (A) in As hyperaccumulating periphytons (in red) and in non-accumulating ones (in green). LDA scores at different taxa levels until the order.

The diversity of microbial communities was studied in the As hyperaccumulator periphytons from area BC2 (TBC2-1 and TBC2-2) and in two non hyperaccumulator periphytons, located at Huatajata and Cohanna Bay (BC3) by 16S rDNA analysis. Microalgae were also indirectly assessed by the genes of chloroplasts. Periphytic communities were dominated by oxygenic phototrophs (including *Cyanobacteria* and microalgae) and by *Proteobacteria* (Supplemental Fig. S5). Two non-accumulating periphyton (HU, BC3) and one hyperaccumulating periphyton (TBC2-1) were dominated by photosynthetic primary producers, and the other hyperaccumulating periphyton (TBC2-2) by heterotrophs. Regarding this, no relationship could be established with As hyperaccumulation. Nevertheless, while non-accumulating periphytons were dominated by *Cyanobacteria*, As hyperaccumulator periphytons mainly harbored microalgae as oxygenic phototrophs. The differential analysis (Lefse, Fig. 7) performed on the basis of microbial community composition highlighted *Cyanobacteria* as significantly more abundant in non-accumulator periphytons. On the contrary, few taxa were specifically more abundant in the As hyperacculator periphytons. Those taxa include OTUs belonging to the *Opitutataceae* (*Verrucomicrobia*) and *Porphyromonadaceae* (Bacteroidetes) families. In addition, the periphyton from TBC2-2 was dominated by *Betaproteobacteria*, with two dominant OTUs related to the *Comamonadaceae* family, affiliated with *Rhodoferax* sp., a purple non sulfur bacterium, and with *Acidovorax* sp., a heterotroph denitrifying bacterium.

## Discussion

### As levels in lake waters

Dissolved As content in Lake Titicaca was around the WHO limit for drinking water (10 µg L^−1^), with some variations (5.7 to 14.9 µg L^−1^). This is consistent with the relatively high geogenic As background reported in the Andes^3^. The slight downstream decrease in dissolved As from the Katari River to the Cohana Bay also suggests anthropogenic As input from the Katari River, like other contaminants brought from upstream urban and mining areas into the lake^29^. Given the wide extension of the lake, additional sources including other tributaries and hydrothermal sources are likely^3^. The results for different periods at BC2 and BC3 did not show a simple relationship between dissolved As content and the lake level. The variations in As content may be partly linked to different partitioning between dissolved and solid phases, with a potential role of the periphyton in As trapping (discussed below). In the Uru Uru area, a strong decrease in As content in water was found between upper and lower part of the lake and reflects inputs from As mining and also scavenging of As by coprecipitation with Fe. Both As(III) and As(V) forms were detected in Lakes Titicaca and Uru Uru, and the proportions were not simply controlled by E_h_ and pH conditions. Possible additional factors controlling As speciation include (1) kinetics effects, since As redox reactions are slow^23^ and the system is subjected to daily variations of redox conditions^19,24^, and (2) complexation reactions of As with iron and dissolved organic matter^25,26^.

### As transfer in totora and non-accumulating periphyton

In both lakes, As transfer in totora shoots was extremely low, with As BAF_totora_ between 0.002 to 0.031. As transfer in periphyton was much higher, with BAFs_periphyton_ from 1281 to 11,962 for the non-accumulating periphyton. Note that BAF_totora_ and BAF_periphyton_ cannot be compared directly because they were calculated versus the sediment and filtered waters, respectively. If the formula for periphyton was used for totora shoots, BAFs would range from 19 to 101, which is still one to three orders of magnitude lower than the periphyton. Thus, based on these BAFs, the periphyton definitely accumulates more As than totora plants. The periphyton from Lake Uru Uru was in the lower end, with BAF_periphyton_ of 1530. These data were compared with several representative studies, from which BAFs could be calculated (Fig. 2, Supplemental Table S6). BAFs were in the same range as observed for natural assemblages of microalgae and bacteria (94 to 27902) and green macroalgae (152 to 13,910). Aquatic plants showed a wider range of BAFs (177 to 46,833).

The absence of periphyton on totora stems at the downstream part of Lake Uru Uru (point UUH) could be due to Cu toxicity^34^, since dissolved Cu was high at this point (45 ± 2 µg L^−1^) compared to the upper part of the Lake Uru Uru (UU12, <0.2 µg L^−1^) and to Lake Titicaca (<1 µg L^−1^).

### As speciation and distribution in non-accumulating periphyton

**Figure 8:**
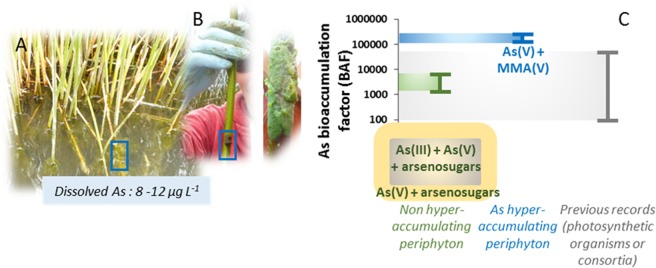
Synthesis of the results. (**A**) Photograph of totora plants showing periphyton attached to the stems in their submerged part. (**B**) Example of periphyton adhering on the stems. (**C**) Comparison of the bioaccumulation factors for the two types of periphyton and previous records, and synthesis on speciation results.

EDTA is assumed to remove cations present in the extracellular compartment. In the present study, EDTA extracted 34 to 76% of total As, and Fe and Mn as well. These data suggest partial sorption of As on Fe and Mn oxyhydroxides present in the extracellular matrix of the periphyton (entrapped particles or biomineralizations). A similar sorption was suggested by Robinson *et al*.^35^ for aquatic plants enriched in Fe and As, growing in As-rich water, leading to a BAF up to 46,833. The comparison of As speciation before and after EDTA extraction that could be quantified for BC4 and BC5 sites showed that in this non hyperaccumulating periphyton, extracellular As was essentially present as As(V) and arsenosugars, and that the intracellular pool contained both As(III), As(V) and arsenosugars (Fig. 8). In both prokaryotes (e.g., *Cyanobacteria*) and eukaryotes (e.g., microalgae), As(V) enters the cells via phosphate transporters^36^. In the present study, phosphorus was not too high (P and PO_4_^3−^ content in filtered water <0.05 and <1.4 mg L^−1^, *i.e*., P < 1.6 µmol L^−1^, Supplemental Table S2, compared to P > 0.1 mg L^−1^ for hypereutrophic lakes). As was between 0.11 and 0.19 µmol L^−1^, so the P/As ratio was less than 8.4. Given this relatively low value, As(V) may compete against P for entry inside cells. As(III) moves across the plasma membrane via aquaglyceroporins and hexose permeases^36^, so its entry does not depend on P nutrition. Algae, microalgae, *Cyanobacteria* and bacteria use different mechanisms to detoxify As(V) and As(III), depending on the contamination level, the As(III)/As(V) ratio, nutrient status and medium conditions (aerobic/anaerobic, euphotic/disphotic). These mechanisms include the oxidation of As(III), the reduction of As(V) into As(III) and its efflux, the formation of methylated As, and the formation of arsenosugars (see reviews for algae^37^, microalgae^36^, *Cyanobacteria*^38^ and bacteria^39^). As(V) has been identified as the major As species in microalgae and *Cyanobacteria* in many studies^36^. As(III) is generally less concentrated because it is excreted^36^, so efflux processes compensate influx. As(III) can also be detoxified inside the cell by vacuolar sequestration and chelation by GSH or phytochelatins^36^. In the present study, no As(III)-thiol complexes were detected. Arsenosugars are the predominant arsenic species in marine algae and they have been found in freshwater microalgae^40^ and *Cyanobacteria*^38^, but there is no evidence of their production by bacteria^38^. In algae, the production of arsenosugars is assumed to be a step subsequent to the methylation of As, and arsenosugars could be precursors or arsenobetaine^38^.

### Evidence of extreme As accumulation in periphyton and possible mechanisms

The periphyton at the external fringe of Cohana Bay (BC2) exhibited extreme As BAFs (up to 245,138). This was observed at three sampling points (BC2, TBC2-1 and TBC2-2 covering a distance of about 300 m). As content was quite variable between replicates and also at the microscopic scale for a given sample, which may be related to the natural heterogeneity of the assemblage. To our knowledge, this is the first time that such high As BAFs have been observed. Previous observations of high As contents in microalgae, microbial mats and aquatic plants were always found in As-contaminated contexts (90 µg L^−1^ to 150 mg L^−1^ As, compared to 8.0 to 14.9 µg L^−1^ in this study, i.e., one to four orders of magnitude higher (Supplemental Table S6). Site BC2 was comparable to the other sites of Lake Titicaca (BC3, BC4, HU) for the measured chemical and physical variables and for As concentration. Therefore the strong hyperaccumulation observed at BC2 is likely biologically controlled and related to certain specific organisms or assemblages. Given the relatively low As content in the water compared to As-contaminated sites, this process cannot be considered to be a response to high As exposure. In the present study, phosphate was below the detection limit (i.e., <1.6 µmol L^−1^) at all the lake sites. A more sensitive method to measure phosphate levels in the lake could help to test whether As hyperaccumulation is related to P nutrition. No EDTA extraction was performed on the As hyperaccumulating periphyton, so the localization of As (intra or extracellular) remains unclear. The XANES results showed the presence of As(V) and MMA(V) in this As hyperaccumulating periphyton (Fig. 8). As(V) was likely present as a mixture of weakly and more strongly bound As complexes. EDTA extractions suggested that Fe/Mn oxides and Ca carbonates could be important sorbing phases for As. Ca carbonates are synthesized by *Characea* and also by *Cyanobacteria*^41^. In previous studies on As-rich photosynthetic organisms and natural assemblages, the main As species included As(V) and As(III) inorganic phases (Supplemental Table S6). For example, mixed As, Fe(III) oxyhydroxides coprecipitates were observed in microbial mats, in the transition zone between reduced and oxidized AMD^42^. Iron oxidizing bacteria were identified, and the As/Fe molar ratios in these extracellular precipitates ranged from 0.4 to 0.7^42^. Aquatic plants growing in As-rich water were also found to contain extracellular As, Fe-rich precipitates^35^. The presence of significant amounts of MMA(V) found in the present study suggests a detoxification mechanism by methylation^36,43^. Further studies by HPLC-ICP-MS should be performed to confirm the presence of this species, since the energy shift between the spectra for MMA and As(V) is small (Supplemental Fig. S4).

Concerning the fate of As present in the hyperaccumulating periphyton after decomposition, the top layer of the sediment at BC2 was not enriched in As compared to the lower layers at BC2 and to the top layers in the other sites. Thus, the sedimentation of dead periphyton biomass does not constitute a significant input of As in the sediment. A possible reason is that the periphyton biomass is minor compared to the other organic and inorganic inputs in the sediment (i.e., totoras biomass which has low As levels).

Taxonomic analyses of the periphyton revealed differences at different taxon levels between the non-accumulating and As hyperaccumulating periphyton, but also considerable variability in composition between the periphyton from TBC2-1 and TBC2-2. *Acidovorax*, a nitrate reducing Fe(II)-oxidizing strain, was identified at TBC2-2. This strain has been shown to precipitate a variety of Fe oxyhydroxides and Fe phosphate in its periplasm and extracellular compartment^44,45^. The ability of *Acidovorax* to sorb As by the formation of inner-sphere complexes at the surface of extracellular Fe oxyhydroxides was shown by Hohmann *et al*.^45^. These authors suggested that exopolysaccharides might also be involved in As immobilization. It would be interesting to test the ability of this strain to accumulate As in moderate As and low Fe conditions such as those prevailing in Lake Titicaca, since previous studies were performed in high Fe and As conditions. However, this strain cannot explain As hyperaccumulation since it was not significantly present at TBC2-1.

This taxonomic analysis is unable to identify an As hyperaccumulator because, to our knowledge, the As bioaccumulation observed in this study has never been reported for any type of organism. More studies are necessary to understand the mechanisms taking place in these As hyperaccumulating periphytons. The As/P competition for uptake and transformations should be investigated *in vitro*. This could be coupled with metagenomics studies targeting already known As transformation genes, As speciation and microbial isolation. These periphytons appear to be excellent biological models for investigating As accumulation in a biological matrix. As hyperaccumulation could result more from a consortium of organisms than in a single organism.

### (Eco) toxicological implications

In terms of (eco)toxicological implications, this study raises the question of the possible transfer of As in the food chain. To our knowledge, there is no report of high As in fishes from Lake Titicaca. Contrary to mercury, As is not known for bioamplification but rather for biodiminution at increasing trophic levels^10^. The main reason is the ability of many organisms to efflux or excrete As, and also to oxidize it into As(V), which is less bioavailable than As(III). Thus, drinking water is in general the major source of As exposure for humans^2^. Concerning the possible exposure of cattle to As through the use of totora shoots as fodder, the daily consumption of 20 kg DW of stems containing 0.6 mg kg^−1^ As would provide 12 mg As. The presence of 0.1% (in weight) of periphyton (estimated from our sampling) would provide 40 mg As in the case of a hyperaccumulator (2000 mg kg^−1^ As) and 0.6 mg As in the case of a non-accumulator (30 mg kg^−1^ As). In parallel, the daily consumption of 60 L of water containing 10 µg L^−1^ by cattle would provide 0.6 mg As. Therefore, in all cases the amount of As provided by fodder is higher than from the consumption of water, and the main contributor is periphyton when it hyperaccumulates As. These simple calculations of total As should be supplemented by the assessment of As bioaccessibility in the biomass.

### Engineering implications

The discovery of As hyperaccumulation in periphyton from water with a moderate As level opens promising perspectives for the treatment of As contaminated waters. Indeed, chronic exposure to As through drinking water is a major public health problem worldwide, affecting hundreds of millions of people, the majority of whom are exposed to moderate As levels^46^. The removal of As from drinking water is costly, so identifying low cost remediation alternatives would be highly beneficial for low income local populations of many areas of the world like that of Lake Titicaca^7^. Regardless of the mechanism (active or passive, intra or extracellular), living organisms capable of BAFs > 100,000 are potentially very interesting for achieving this. Various technologies could benefit from these organisms, including constructed wetlands, rhizofiltration and biofiltration systems. In constructed wetlands, metals are mostly accumulated in the sediment, both by sedimentation of organic flocs and by fixation in the rhizosphere^15^. Microorganisms, biofilms and periphyton play a key role in these processes. Rhizofiltration uses plants floating on a contaminated stream, and the removal of contaminants is performed by root uptake or sorption on roots. This technique has been applied for As removal^47^. Finally, systems using bacteria^48^ or microalgae-bacteria consortia^49^ (so called bioremediation) are currently being studied for contaminant removal. Further research is needed to identify As hyperaccumulating species or consortia and the conditions required for this phenomenon to occur. Moreover, it is necessary to evaluate their cultivation, their efficiency and the kinetics of As accumulation in various environmental conditions.

## Methods

### Description of the sites

Lake Titicaca and Lake Uru Uru are part of the same high altitude endorheic system (Fig. 1). They are subjected to high UV and low O_2_ conditions, and slight to moderate salinity. Both lakes are connected by the Desaguadero River, flowing from Titicaca to Uru Uru, which is connected downstream to Lake Poopo. Lake Titicaca covers 8372 km^2^ and contains a large, deep northern basin (Lago Mayor, average depth 107 m, max. depth 284 m), and a shallow sub-basin to the south (Lago Menor, average depth 10 m, max. depth 40 m). The shallowest zone of Cohana Bay is colonized by totoras and receives water from the Katari River, which drains the densely populated area of El Alto^29^. Sampling was done along a transect in Lago Menor from the Katari River estuary (BC5) across Cohana Bay to Huatajata (HU), and upstream in the Katari (TA) and Palina (PU, PA) tributaries (Fig. 1, Supplemental Table S1). The lake shores and Cohana Bay are colonized by totoras and *Characeae*, a green macroalgae encrusted with calcium carbonate biominerals. *Characeae* are even more represented in the open part of Lago Menor. Point BC2 at the entry of the bay is close to the ecological transition between a zone with totora and *Characeae* and the open lake where only *Characeae* are present.

The Titicaca area is characterized by a wet and a dry season, resulting in small variations of the level of Lake Titicaca (Supplemental Fig. S1). Several field samplings were performed, as the levels of the lake rose (PB1, PB2, PB5) and fell (PB3, PB4) (Supplemental Fig. S1). Campaign PB2 (April 2015) on Lake Titicaca took place during a massive algal bloom, creating a partial anoxica in the water column^24^. Campaign PB5 took place during the rainy season and after a rainfall event, and the lake water close to the Katari outlet (BC4) was highly turbid and brownish. In addition to the lake water, one sample of rainwater was collected at Huatajata.

Lake Uru Uru (Fig. 1) is impacted by intense mining and smelting activities (mostly Sn and Sb, but also Ag, Au, Cu, Zn, Bi, W)^19^. It is very shallow (average depth 0.7 m) and is subject to considerable evaporation due to the semi-arid context. The Huanuni River, one of the tributaries, is strongly impacted by acid mine drainage (AMD). Three sampling points were selected, based on a previous work on methylmercury distribution in the area^19^: UU12, which is in the largest part of Lake Uru Uru (corresponding to a point 14.4 km from the outlet of the Tagarete River, which carries untreated wastewater from Oruro area, a mining and smelting zone, into the lake); the AMD tributary Huanuni River (RH); and UUH in the narrower part of the lake, downstream of Rio Huanuni (Fig. 1, Supplemental Table S1). Both sampling points in the lake (UU12 and UUH) are colonized by totora, while *Characeae* can be found at UU12. All the samples from the Uru Uru area were collected during campaign PB5, during the wet season.

### *In situ* measurements and sampling

The physico-chemical parameters of the water were measured *in situ* using a submersible probe. Details on sampling of the various compartments (water, suspended particulate matter, rain water, surface sediment, totora plants and periphyton) are provided in Supplemental Methods. For the periphyton, EDTA extraction was performed to separate the intra and extracellular compartment, as described previously^50^.

### Analyses

#### Elemental concentrations

Solid samples (sediment, plant and periphyton) were freeze dried, ground in an agate mortar and digested following the protocol described in Supplemental Methods. Major and trace element concentrations in solid samples after digestion and in filtered water were measured by ICP-AES and ICP-MS, respectively. All the experimental details are provided in Supplemental Methods. All elemental concentrations in solid samples were expressed on a dry weight basis (DW).

The As bioaccumulation factors (BAF) were calculated as follows:

$${{\rm{BAF}}}_{{\rm{periphyton}}}={[{\rm{As}}]}_{{\rm{periphyton}}}({\rm{in}}\,\mu g\,{{\rm{kg}}}^{-1}{\rm{DW}})/{[{\rm{As}}]}_{{\rm{filtered}}{\rm{water}}}({\rm{in}}\,\mu g\,{{\rm{L}}}^{-1})$$

$${{\rm{BAF}}}_{{\rm{totora}}}={[{\rm{As}}]}_{{\rm{stem}}}({\rm{in}}\,\mu g\,{{\rm{g}}}^{-1}{\rm{DW}})/{[As]}_{{\rm{sediment}}}({\rm{in}}\,\mu g\,{{\rm{g}}}^{-1}{\rm{DW}})$$

#### Other chemical analyses

The methods used to determine anions, dissolved organic carbon (DOC), and dissolved hydrogen sulfide (H_2_S + HS^−^) in water samples, and acid volatile sulfides (AVS), simultaneously extracted metals (SEM), and loss on ignition (LOI) are described in Supplemental Methods.

#### X-ray absorption spectroscopy

As K-edge XANES measurements on the periphyton were performed at the beamline FAME (BM30B) at the European Synchrotron Radiation Facility (ESRF) in Grenoble, France. Experimental details regarding data acquisition and data treatment are provided in Supplemental Methods. Several As reference spectra were provided by colleagues including arsenosugars (glycerol sugars extracted from brown algae *Fucus vesiculosus*), monomethylarsenate (MMA(V)) and dimethylarsenate (DMA(V))^51,52^, both in solid state, sorbed As(V) species and As^III^-glutathione^53^, and thioAs(V) species^54^ (Supplemental Methods).

#### Taxonomic composition of the periphyton

The methods used for species identification in periphyton by optical microscopy and DNA sequencing are described in Supplemental Methods.

## Supplementary information

Supplementary info

## Data Availability

Site locations and chemical raw data are provided in Supplementary Information. XAS data are available upon request to the authors. The sequence data have been submitted to the Sequence Read Archive of the National Center for Biotechnology Information and are available under accession number PRJNA508881.
