# Supplementary material for: Extreme Arsenic Bioaccumulation Factor Variability in Lake Titicaca, Bolivia
Source: Sci Rep. 2019 Jul 23;9:10626. doi: 10.1038/s41598-019-47183-8 (PMC6650431; doi:10.1038/s41598-019-47183-8)
Supplement: Supplementary file 1 — Supplementary info [file 41598_2019_47183_MOESM1_ESM.pdf]

Supporting information

Extreme Arsenic Bioaccumulation Factor Variability in Lake Titicaca, Bolivia

Géraldine Sarret, Stéphane Guédron, Dario Acha, Sarah Bureau, Florent Arnaud-Godet, Delphine Tisserand, Marisol Goni-Urizza, Claire Gassie, Céline Duwig, Olivier Proux, Anne-Marie Aucour

This file consists of 16 pages, and includes 5 figures and 7 tables.

1. Supplemental Methods

1.1. Sites description

Table S1: Sampling locations

Area	Location	Distance from katari river inlet (km)	Sam-pling code	Sampled during campaign	GPS coordinates
Tributaries of Lake Titicaca	Palina River at Puchukollo (dowstream the WWTP)	50.9	PU	PB5	16°32'0.64"S 68°15'5.52"W
	Palina river	34.5	PA	PB5	16°32'20.98"S 68°24'14.62"W
	Downstream confluence of Katari and Palina River	22.9	TA	PB5	16°31'15.31"S 68°30'18.30"W
	Katari River before inlet in Cohana bay	2.0	BC5	PB5	16°22'15.11"S; 68°39'6.58"W
Lake Titicaca (Lago menor)	Cohana Bay	3.0	BC4	PB5	16°21'56.88''S; 68°41'43.98''W
	Cohana Bay	5.7	BC3	PB1,2,5	16°21'48.06''S; 68°43'16.14''W
	Cohana Bay	8.2	TBC2-2	PB4	16°20'33.1''S; 68°44'3.6''W
	Cohana Bay	8.3	BC2	PB1,2,3,4	16°20'40.13''S; 68°44'8.12''W
	Cohana Bay	8.4	TBC2-1	PB4	16°20'41.3''S; 68°44'9.1''W
	Transect across lago menor	9.0	T3	PB2,3	16°20'17.94''S; 68°44'9.48''W
	Transect across lago menor	15.2	T2	PB2,3	16°17'14.40''S; 68°43'2.88''W
	Transect across lago menor	20.2	T1	PB2,3	16°14'45.24''S; 68°42'3.78''W
	Huatarata	23.9	HU	PB4,5	16°12'47.13''S; 68°41'33.25''W
Tributary of Lake Uru Uru	Huanuni River (acid mine drainage)		RH	PB5	18°9'57.82"S 66°59'12.01"W
Lake Uru Uru	Lake Uru Uru		UU12	PB5	18°09'15.62'' S 67°04'46.76''W
	Uru Uru outlet, downstream Huanuni River		UUH	PB5	18°21'42.52"S 67°2'46.18"W

Sampling campaign	Date
PB1	Feb, 3, 2015
PB2	Apr., 15, 2015
PB3	Oct., 10, 2015
PB4	June, 6, 2016
PB5	March, 20, 2017
PB5	April, 16, 2017

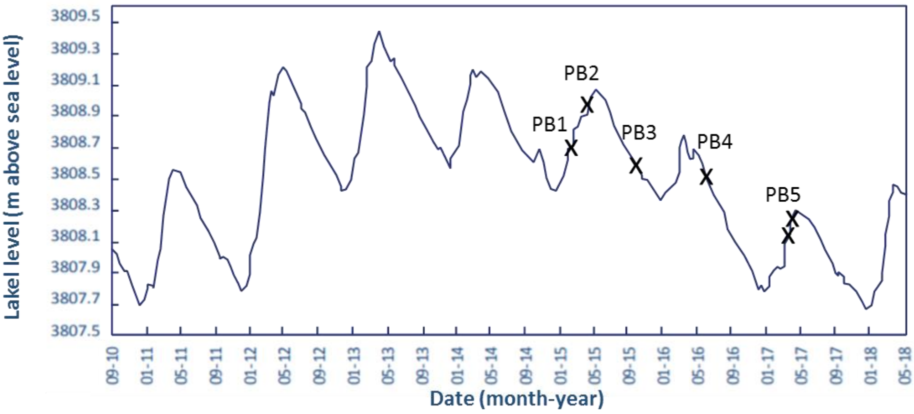

Figure S1. Dates of the five sampling campaigns and corresponding level of the Lake Tititcaca at Huatarata (source: http://www.senamhi.gob.bo/).

2. In situ measurements and sampling

Physico-chemical parameters (temperature, pH, conductivity, redox potential (Eh, also called oxidation reduction potential (ORP), dissolved oxygen and salinity) were measured in situ using a submersible multiparameter probe (HANNA). Lake water samples were all collected at 0.2 m depth, in PTFE bottles previously cleaned with acid, and filtered at 0.22 µm using Sterivex PVDF filters. Some water samples were passed through an As speciation cartridge (Metalsoft) that retains As(V). The As(V) removal efficiency of the cartridge was checked with synthetic As(V)-As(III) solutions and was between 92 and 100%. Filtered water was then placed in acid washed 15 mL polypropylene tubes. All water samples were acidified with HCl (0.5%, v/v) and kept at 4°C until analysis. For anions analyses, filtered water was placed in 15 mL PP falcon tubes, frozen and kept at -18°C until analyses. For total organic carbon (TOC or NPOC for non purgeable organic carbon) analyses, filtered water was placed in glass dark tubes previously cleaned by calcination, and stored at 4°C until analyses. One sample of suspended particulate matter was obtained by filtration under vacuum of 2 L of lake water. One sample of rain water was collected at Huatajata station.

Surface sediments were collected using a gravity corer as described in (Guedron et al., 2017). Cores were immediately extruded and sediment was collected in 50 mL falcon tubes. For AVS, the tubes were filled to the top to avoid the contact with air. Totora plants were collected, rinsed with ultrapure water, directly placed at 4°C for maximum 12h, frozen and freeze dried. Periphyton was collected on totoras stems, at a depth of 0 to 2 m. Periphyton PB4-HU was collected on glass slides placed at 0.5 to 3.5 m depth for two months. EDTA extraction was done as described (Meylan et al., 2004). Briefly, about 10 mL of fresh periphyton was placed in 500 mL teflon bottle containing 4 mM EDTA for 10 min. The periphyton was then soaked and an aliquot of solution was filtered and kept for analysis. After sampling (and EDTA extraction for some of them), all periphyton samples were placed at 4°C for maximum 12h, frozen and freeze dried.

1.2. Arsenic and major elements (Fe, Mn, Ca, K, P, S, Al) in solid and water samples

The samples of plant material and sediment (between ca. 20 and 300 mg) were digested using high purity reagents (concentrated sub-boiled HNO_3, HF and HCl, HBr seastar^{TM}, suprapur 30% H_2O_2) at 110°C. All the samples were digested in 6:2 ml HNO_3:H_2O_2. The plants were further digested in 6:2 ml HNO_3:H_2O_2 and 2:0.5 ml HNO_3:HF, the sediment in 3:0.75 ml HNO_3:HF and 1:2 ml HNO_3:HCl.

Concentrations of As, Mn, Fe were measured by ICP-MS (Agilent 7500 CX) at ENS Lyon for the filtered waters and for the plant and sediment digests. The solutions run on ICP-MS were prepared in HNO_3 0.5 N, spiked with 2 µg L^{-1} indium, which is used as internal standard. Arsenic was measured on ^{75}As with H_2 reaction mode and He collision mode, ^{56}Fe with He (collision mode), ^{55}Mn with He (collision mode) and Ar gas carrier. ^{75}As measurements with H_2 or H_e in general agreed well; there is also in general a very good consistency between ^{55}Mn measurements made with He (collision cell) and Ar. The detection limit of As, Fe, Mn in the solutions run on the ICP-MS was ca. 0.1 µg L^{-1}. Filtered water samples were diluted 1:2 as well as 1:5 in HNO_3 0.5 N medium; thus the final detection limit was 0.2 µg L^{-1}. Measurements were made in the range 1-40 µg L^{-1} for As, 1-300 µg L^{-1} for Fe and Mn. Results obtained for both dilutions (1:2 and 1:5) agree well. For plant, sediment and periphyton samples, the evaporated digest was first taken in 2 mL; an aliquot of the digest (50-500 µL depending on the digest concentration) was diluted in 10 mL HNO_3 0.5 N (dilution 1:200- 1-:20) so that the final concentration of the solution run on ICP-MS falls within the ranges given above (if this is not the case, further appropriate dilution was made). Repeated analysis of filtered water and solid samples within and between analytical sessions gives a precision of ± 5%.

Major elements were measured in the filtered solutions (Ca, K, P, S, Al) and in the plant and sediment digests (Fe, Mn, Ca, K, P, S, Al) by inductively coupled plasma spectrometry – atomic emission spectrometry (ICP-AES) using an Agilent 720 ES at ISTerre. Arsenic was also measured by ICP-AES when the concentration of the solutions run on ICP-AES was above detection limit of 50 µg L^{-1}. For the filtered solutions, there was no dilution before analysis. For the solid samples, the evaporated digest was diluted in 50 mL with 2% HNO_3. Calibration was performed by dilution of standard solutions at 1000 µg L^{-1}. A set of water and solid samples has been measured independently by ICP-AES and ICP-MS for As, Mn, Fe and there was in general a good agreement (within a few %) between both measurement techniques. The detection limit for each element in the solutions run on the ICP-AES and the corresponding concentrations in mg kg^{-1} of dry solid material before digestion are given below:

Limit of quantification (LQ) for the ICP-AES measurements

	As	Fe	Mn	Ca	P	S	K	Al
LQ, mg L^{-1}	0.05	0.05	0.005	1	0.09	0.2	0.5	0.2
LQ, mg kg^{-1} of dry solid*	50	50	5	1000	90	200	500	200

* 50 mg of solid diluted in 50 mL solution

For both analytical techniques, solid reference materials (JSd1, MESS3, BRC679) digested using the protocol described above and solution standards (Roth) were measured at each session and at different times during the session. Measured values agreed with certified values within less than 5 %. To further test the quality and consistency of the analyses, sediment and periphyton samples were digested in parallel in the two laboratories, and analyzed for As, Fe and Mn both by ICP-AES at ISTerre and by ICP-MS at ENS Lyon. Results were in very good agreement, with a few % difference.

1.3. Content in anions, dissolved organic carbon, dissolved hydrogen sulfides in water samples

Anions were measured at IGE (OSUG-Grenoble) by ionic chromatography using a 332 Metrohm apparatus. External calibration was done using monomolecular standards at 1000 mg L^{-1}, with various dilutions to cover the range of concentrations. The accuracy, evaluated on a multimolecular standard (Carl Roth 2668.1), was between 3 and 11%. The drift of the machine during the measurement session, corrected with the repeated analysis of a PO_4^{3-} standards, was between 3 and 13%.

Dissolved organic carbon (DOC) was measured with a TOC-VCSN analyzer from Shimadzu. The DOC is transformed into CO_2, and detected by infrared. External calibration was done using certified standards at 100 mg L^{-1} (ChemLab). The accuracy, evaluated on a certified standard, was between 0.8 and 11%. The precision, evaluated by repeated measurements on the same sample, was < 2.5%. The drift of the machine, corrected with the repeated analysis of standards during the measurement session, was between 1 and 11%.

Samples for dissolved hydrogen sulfides (ΣH_2S) were collected directly into a degassed vacuum container, previously filled with 0.5 mL of a diamine mixture prepared as recommended (Reese et al., 2011). The method used is a modification of the previously described ones (Small and Hintelmann, 2007; Small and Hintelmann, 2014). It determines the concentration of H_2S and HS^- (converted into H_2S by the reagents). Briefly, 20 µL of the sample with the diamine mixture was injected into an Agilent 12600 HPLC with a Poroshell 120 EC-C18 Agilent column with a mix of 20% acetonitrile, 18% methanol, 20% sodium acetate buffer (pH 5.2, 0.05 mM) at 35°C and 1.1 mL min-1. Concentrations were determined using the Radiello® calibration solution for H_2S Code 171.

1.4. AVS, SEM and loss on ignition for sediment samples

Acid volatile sulfides (AVS) measurements have been performed using miniaturized and duplicate apparatus developed at ISTerre adapted from (Allen et al., 1993). To avoid sample oxidation, samples were kept in flask filled until the top and were kept under N_2 atmosphere once at the laboratory. AVS extraction and quantification consisted on degasing AVS from the wet sediment by acidifying with HCl 6N and bubbling under N_2 to generate H_2S gaz. Aliquots of wet sediment ranged from 30 to 600 mg, in order to match the calibration range. H_2S gaz was then trapped in a sodium hydroxide solution to form a stable molecule finally quantified by a spectrophotometric method with the generation of methylene blue complex referred as Cline's method (Cline, 1969). AVS results are expressed in µmol g^{-1} DW after freeze drying an aliquot of fresh sediment to determine its water content. Calibration was performed using a solution prepared with Na_2S, $9H_2O$ reagent and titrated by an iodometric method (Fishman and Friedman, 1989). Measurement accuracy was determined by analyzing 2 times several samples, and ranged from 8 to 16%. The simultaneously extracted metals (SEM) including Cd, Cu, Ni, Pb, Zn, As and Ag were analyzed by ICP-AES (Agilent 720 ES) at ISTerre following the protocol by Di Toro et al. (Di Toro et al., 2005). Standards were prepared with monometallic ICP standard solutions at 1000 mg L-1 diluted in HCl 6N in order to avoid any matrix effect. The machine drift was corrected based on the regular analysis of a standard during the sequence. It was always <5%. Results were corrected from the blanks measured in the same conditions as the samples. The organic content of the sediments was evaluated by the loss on ignition (LOI), which is the percentage of weight lost after 3 hours at 550°C.

1.5. XAS spectroscopy on periphyton samples

As K-edge XANES measurements on the periphyton were performed at the beamline FAME (BM30B) at the European Synchrotron Radiation Facility (ESRF) in Grenoble, France, operating in 7/8 filling mode, with a current between 160 and 200 mA. The monochromator was a Si(220) double crystal with sagittal focusing. Spectra were recorded in fluorescence mode using a 30-element Canberra Ge detector, at 10°K using a He cryostat. As reference spectra included arsenopyrite $FeAs^{III}S$, arsenic trisuflide ($As^{III}_2S_3$), As^{III} oxide ($As^{III}_2O_3$), sodium arsenite ($NaAs^{III}O_2$), As^{III} sorbed on ferrihydrite, sodium arsenate, As^V sorbed on goethite, As^V sorbed on ferrihydrite and As^{III}-glutathione (As^{III}-GSH) (last four provided by Raoul Marie-Couture), arsenosugars (glycerol sugars extracted from brown algae Fucus vesiculosus), dimethylarsenate (DMA(V)) and monomethylarsenate (MMA(V)) provided by Iris Koch, and As^V sorbed on calcite, and mono-, di- and tetra-thioAs provided by Andreas Scheinost and Britta Planer-Friedrich.

It was not possible to ship frozen periphyton samples from Bolivia to France, so spectra were recorded on freeze-dried samples. To ensure that this treatment did not alter As speciation, a test experiment was conducted on fresh periphyton collected in France. Periphyton samples were collected in Lake la Batie (le Versoud, France, 45°13’46.646’’N 5°51’2.487’’ E). They were incubated for 3 h in 500 mL bottles containing the lake water spiked with 1 mg L^{-1} As^{III} ($NaAsO_2$) or As^{V} ($HAsNa_2O_4$) at pH 6.9 (pH of the lake). The periphyton was then collected and pressed to remove the water, half was frozen and half was freeze-dried. As K-edge XANES spectra were recorded on the four samples. The spectra recorded in frozen hydrated and freeze-dried state were very similar (Figure S2A), and linear combination fits provided similar results, with 3 to 5% difference in the percentages (Figure S2 B-C). So it was concluded that freeze drying does not alter the speciation of As present in the periphyton.

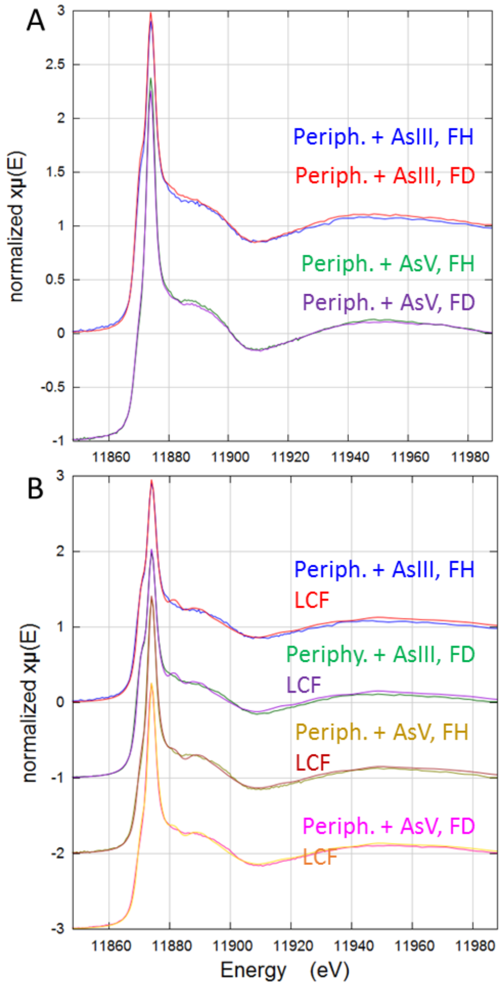

C

As species (%)		
AsIII-O	AsV	R factor
0.54	0.50	0.0025
0.59	0.48	0.0023
0.41	0.65	0.0035
0.44	0.62	0.0033

Figure S2: Comparison of S K-edge XANES spectra for periphyton samples from La Batie after incubation in 1 mg L^{-1} AsIII or AsV, in freeze dried (FD) and frozen hydrated (FH) state (A). B, Linear combination fits, and C, percentages of AsIII and AsV species obtained from the LCFs.

1.6. Diversity of periphyton communities by DNA sequencing

DNA extractions were performed from lyophilized samples. Prior to extraction with DNeasy PowerSoil Kit (MoBio), samples were hydrated with 200µL de Tris EDTA pH 8 and added to the Powerbead tube. A grinding step (2 times 30s, 5000 RPM) in a Precellys instrument (Bertin ins.). DNA concentration was measured in a microplate reader (BioTek) using the Quant-iT dsDNA Assay Kit, broad range (Thermo Fisher Scientific). Bacteria and archaeal communities’ composition was determined based on the V4 16SrDNA gene polymorphism. PCR reactions contain 1x AmpliTaq Gold 360 Master Mix – (Thermo Fisher Scientific), 0.5µM of each primer 515F (CTTTCCCTACACGACGCTCTTCCGATCTGTGYCAGCMGCCGCGGTA) and 928R (GGAGTTCAGACGTGTGCTCTTCCGATCTCCCCGYCAATTCMTTTRAGT) (Wang and Qian, 2009) and 5ng of DNA. Thermal cycling was carried out in an AmpGene 9700 (ABI) as follows: 10 min at 95°C, 30 cycles at 95°C for 30 s, 60°C for 30 s and 72°C for 40 s and a final extension for 7 min at 72°C. Amplicons were sequenced using MiSeq 250-paired technology (Illumina), with V3 kit, in Get-plage sequencing platform (INRA, Toulouse). Data were analysed using FROGS (Find, Rapidly, OTUs with Galaxy Solution) tool (Escudié et al., 2018).

Before statistical analysis, random sampling of filtered data was performed to obtain the same number of reads per sample. Taxonomic biomarkers of As hyperaccumulator periphyton were detected using the LEfSe algorithm (Segata et al. 2012). Briefly,

a non-parametric Kruskal-Wallis (p-value <0.05) sum-rank test was performed to detect taxa with significant differential abundances, followed by a pairwise Wilcoxon test (p-value <0.05) in order to detect biological consistency of biomarkers. Finally, linear discriminant analysis (LDA, threshold of 2) leads to estimate the effect size of each differentially abundant taxon.
Raw sequences were submitted to the National Center for Biotechnology Information Sequence Read Archive under the number PRJNA508881.

2. Supplemental Results

2.1 Filtered lake waters

Table S2. Concentrations in arsenic in filtered lake waters. As SC : arsenic concentration after filtration on As speciation cartridge, which removes As(V). Data for Fe, Mn, Cl^-, S, SO_4^{2-}, ΣH_2S, NO_3^-, PO_4^{3-}, DOC in filtered waters and pH, E_h, salinity are also presented. Numbers in italics and brackets give the standard deviation measured on triplicate samples.

Sampling site	Campaign	As	As SC	Fe	Mn	pH	E_h	DO [(6)]	Salinity	Cl^-	S	SO_4^{2--}	S as	ΣH_2S	S as	NO_3^{--}	PO_4^{2-}	Ca	Na	DOC
		µg L^{-1}	µg L^{-1}	µg L^{-1}	µg L^{-1}		mV	%	g L^{-1}	mg L^{-1}	mg L^{-1}	mg L^{-1}	SO4[2-] %	µM	ΣH_2S %	mg L^{-1}	mg L^{-1}	mg L^{-1}	mg L^{-1}	mg L^{-1}
PU	PB5 (april 2017)	3.6		817	1700					33		169				<LQ	<LQ			
PA	PB5 (april 2017)	8.2		52	716					59		94				<LQ	<LQ			
TA	PB5 (april 2017)	11.0		34	361	7.4	-19.0			59		90				<LQ	<LQ			
BC5	PB5 (april 2017)	9.0	2.5	28.6	269	6.7	-35.0		0.27	33	27.6	68	82			3.1	<LQ	35.8	35.7	12.4
BC4	PB5 (march 2017)	5.7 *(0.2)*	1.8 *(0.1)*	17 *(11)*	339 *(6)*	7.3	21.0		0.27	37 (0.3)	27.3 *(0.3)*	72 *(1.0)*	88			3.4 *(0.2)*	<LQ	44.2 *(0.4)*	40.6 *(0.2)*	6.9 *(0.9)*
	PB5 (april 2017)	8.2 *(11.1)*	4.1	17 *(5)*	450 *(27)*						62.5			1.5 *(0.5)*	0.18			61.4		
BC3	PB1	12.6 *(0.3)*		22 *(8)*	36 *(3)*	7.0	97[(1)]	64	0.56		53.2							70.6	129	
	PB2	5.0				7.1	-325[(2)]	33	0.67	190 *(7)*	65.7 *(0.2)*	184 *(1.0)*		3.2 *(0.2)*	0.16	<LQ	<LQ	50.4 *(0.1)*		13.6 *(1.9)*
	PB5 (march 2017)	12.1 *(0.1)*	5.2 *(0.3)*	44 *(3)*	84 *(4)*	7.8	-170		0.52	112 *(1)*	50.4 *(0.8)*	131 *(1.0)*	87	2.0 *(0.3)*	0.13	<LQ	<LQ	57.7 *(0.8)*	99.4 *(1.1)*	9 *(0.2)*
BC2	PB1	11.3 *(0.5)*		27	3.7	8.2	125[(3)]	97	0.79		95.4							71	247	
	PB2	8.0				7.4	-334[(4)]	14	0.80	213 *(3)*	76.2 *(0.5)*	211 *(1.0)*		2.5 *(0.3)*	0.11	<LQ	<LQ	68.4 *(0.2)*		9.2 *(1.5)*
	PB3	9.0 *(0.1)*		4.4 *(1.2)*	3.9 *(0.2)*	8.2	-59[(5)]	17	0.67	291 *(0.6)*	94.9 *(0.3)*	182 *(0.9)*	87			<LQ	<LQ	58.2 *(0.1)*	217 *(0.8)*	6.8 *(0.4)*
	PB4	14.1		1.3	2.2	7.5			0.74	273 *(0.5)*		262 *(0.5)*				<LQ	<LQ			
TBC2-1	PB4	14.9		2.9	2.9	7.8			0.73	269 *(5)*	90.3	258 *(11)*	95			<LQ	<LQ	50.6	204	
TBC2-2	PB4	14.3		3.8	3.2	7.8			0.74	267 *(1)*	91.5	250 *(4.2)*	91			<LQ	<LQ	50.1	206	
T3	PB3	8.9		6.7	1.3	8.4		106	0.78	273	93.3	267	96			<LQ	<LQ	57.1	213	5.6
T2	PB3	8.2		3.5	0.8	8.3		99	0.79	283	91.5	275	100			<LQ	<LQ	62.1	210	6
T1	PB3	8.5		1.8	0.7	8.5		112	75.00	266	88.4	277	105			<LQ	<LQ	64.5	202	3.9
HU	PB4	13.5		1.8 *(0.3)*	1.1	7.5			0.73	241 *(11)*	88.7 *(0.2)*	253 *(11)*	95			<LQ	<LQ	65.5 *(0.2)*	198 *(0.4)*	
	PB5 (march 2017)	11.7 *(0.6)*	1.5	11.6 *(6.8)*	0.7 *(0.1)*					258	88.8	209	79			<LQ	<LQ	61.8	202	3.6
HU (rain)	PB1	<0.2																		
UU12	PB5 (march 2017)	78.5 *(5.5)*	11.9 *(1.8)*	102 *(9)*	126 *(3)*	7.5	-12		2.11	776 *(43)*	142 *(0.9)*	377 *(28)*	88	5.5 *(0.4)*	0.12	<LQ	<LQ	126 (0.8)	533 *(3.5)*	10.7 *(0.2)*
UUH	PB5 (march 2017)	4.8 *(0.4)*	2.1 *(0.1)*	28 *(5)*	404 *(5)*	8.0	132		2.31	869 *(6)*	169 *(1.2)*	500 *(5)*	99	1.0 *(0.3)*	0.02	<LQ	<LQ	147 (0.8)	588 *(3.8)*	10.6 *(0.6)*
RH	PB5 (march 2017)	84 *(10)*		111478 *(3756)*	21092 *(585)*	3.0	402		1.01	72 *(2.5)*	376 *(1.6)*	1206 *(76)*	107	2.1 *(0.4)*	0.02	<LQ	<LQ	110 (0.5)	33.4 *(0.2)*	

As SC: As content after As speciation cartridge, corresponding to As(III).
(1) value at 0.2 m depth. 88 mV at 0.5 m. -129 mV at 1.0 m; (2) value at 0.2 m depth. -364 mV at 0.5 m. -359 mV at 1.0 m. sampling during an algal bloom; (3) value at 0.2 m depth. 123 mV at 0.5 m. 136 mV at 1.0 m
(4) value at 0.2 m depth. -400 mV at 0.5 m. -379 mV at 1.0 m. sampling during an algal bloom; (5) value at 0.2 m depth. -108 mV at 0.5 m. -137 mV at 1.0 m; (6) DO% : dissolved oxygen saturation %
< LQ: below anion quantification limit (1.4 mg L^{-1})

2.2 Sediments

Table S3. Concentrations in arsenic and major elements (Fe. Mn. Ca. K. P. S. Al) in sediments and suspended particulate matter (SPM). Acid volatile sulfides (AVS) and Fe associated with AVS were also measured at selected sites.

Sampling site	Campaign	Sample	*n*	As µg g^{-1}		Fe %		Mn µg g^{-1}		Ca %		K %		P µg g^{-1}		S %		Al %		AVS µmol g^{-1}		Fe AVS µmol g^{-1}	
				mean	σ	mean	σ	mean	σ	mean	σ	mean	σ	mean	σ	mean	σ	mean	σ	mean	σ	mean	σ
BC5	PB5	sed. 0-5 cm	1	27		4.20		460		0.44		2.26		1799		0.12		9.06		12.1	1	511	7
BC4	PB5	SPM	1	54		4.58	`	585		0.34		2.58		1300		b.d.		8.64					
BC3	PB1	sed. 0-3 cm	1	41		4.34		433		0.86		2.37		1064		3.22		8.79					
		sed. 3-10 cm	1	45		4.18		371		2.70		2.38		913		3.00		8.65					
		sed. 10-20 cm	1	21		4.31		382		2.32		2.41		938		3.04		8.84					
	PB5	sed. 0-3 cm	1	50		4.83		571		0.87		2.69		870		3.28		10.59		55.6		220	
		sed. 3-10 cm	1	43		4.86		484		1.22		2.52		806		3.28		10.04		30.0		166	
		sed. 10-20 cm	1	44		5.10		513		0.75		2.73		747		3.30		10.88		28.1		194	
BC2	PB1	sed. 0-3 cm	1	59		1.40		151		9.94		0.88		1446		1.86		2.74					
		sed. 3-10 cm	1	44		2.24		183		13.62		1.38		682		1.99		4.48					
		sed. 10-20 cm	1	28		1.91		145		14.89		1.26		573		1.81		4.01					
	PB2	sed. 0-3 cm	1	62		1.97		167		1.77		1.13		1614		2.59		4.02					
		sed. 3-6 cm	1	48		2.27		181		2.00		1.30		1383		2.54		4.56					
		sed. 6-10 cm	1	30		2.28		161		13.09		1.42		679		1.97		4.58					
UU12	PB5	sed. 0-3 cm	2	76	8	2.83	0.17	409	19	3.13	0.12	2.07	0.05	938	52	0.80	0.03	6.79	0.40	217.2	0.0	319	
	PB5	sed. 3-10 cm	2	45	0.1	3.03	0.02	430	19	1.69	0.02	2.31	0.08	664	14	0.72	0.03	7.50	0.40	196.8	29.6	347	3
	PB5	sed. 10-20 cm	2	50	4	3.25	0.04	545	9	1.79	0.00	2.33	0.00	602	2	0.75	0.03	7.54	0.11	318.9	5.1	461	110
UUH	PB5	sed. 0-5 cm	2	181	3	4.72	0.11	789	24	3.48	0.50	1.97	0.07	999	1	0.74	0.02	8.35	0.87	129.8	0.87	590	
RH	PB5	sed. 0-5 cm	1	491		4.58		196		0.15		0.91		430		1.98		4.44		32.8		390	18

n : number of samples analysed

2.3 Totora plants

Table S4. Concentrations in arsenic and major elements (Fe, Mn, Ca, K, P, S, Al) in totora samples (in μg g^{-1} dry weight) and bioaccumulation factor (BAF) in shoot for As.

Sampling site	Campaign	Sample	*n*	As		BAF_{totora}	Fe		Mn		Ca		K		P		S		Al	
				mean	σ		mean	σ	mean	σ	mean	σ	mean	σ	mean	σ	mean	σ	mean	σ
BC5	PB5	roots	1	65.0			28081		1314		5395		15305		5350		3296		20542	
		rhizome	1	1.7			1157		177		903		13249		3661		1236		1521	
		shoot	1	0.7		0.026	192		1082		5204		27296		2849		3438		180	
BC3	PB1	roots	1	30.8			1579		208		<1000		23666		932		16227		<200	
		rhizome	1	1.6			85		17		<1000		18328		1928		5524		<200	
		shoot	1-2	1.3	1.0	0.031	<50		394		<1000		10787		585		2263		<200	
BC2	PB1	roots	1-2	46.7	4.3		736		302		1645		25552		1159		12910		<200	
		rhizome	1	0.9			<50		21		<1000		16813		1429		1491		<200	
		shoot	1-2	0.6	0.2	0.010	81		165		<1000		17576		648		2294		<200	
	PB2	shoot	1	167.7			2486		185		7696		8865		1584		15825		4680	
UU12	PB5	roots	3	30.9	3.9		717	83	292	24	2896	889	13806	2529	2080	358	4119	584	832	87
		rhizome	3	1.7	0.1		41	1	64	4	408		4874	166	1027	17	707	56	< 200	
		shoot	3	1.5	0.1	0.019	53	2	636	21	1730		24828	500	1677	49	2984	62	< 200	
UUH	PB5	roots	3	50.2	4.6		13983	649	488	42	3139	591	14347	468	854	51	6042	322	7686	734
		rhizome	3	3.9	0.1		977	21	229	3	1506		6393	276	423	3	1884	143	528	58
		shoot	3	0.4	0.1	0.002	75	10	625	14	2527	805	17044	298	4608	163	<200		181	

n = number of replicates (independent sample collection, digestion and analysis).

2.4 Periphyton

Table S5. Concentrations in arsenic and major elements (Fe, Mn, Ca, K, P, S, Al) versus dry weight in periphyton samples and bioaccumulation factor (BAF) for arsenic.

Sampling	Cam-	Sample	*n*	As		$BAF_{periphyton}$	Fe		Mn		Ca %		K		P		S		Al	
				µg g^{-1}		L g^{-1}	µg g^{-1}		µg g^{-1}				µg g^{-1}		µg g^{-1}		mg g^{-1}			
site	paign			mean	σ		mean	σ	mean	σ	mean	σ	mean	σ	mean	σ	mean	σ	mean	σ
BC5	PB5	periphyton	2	58.3	6.9	6459	42912	4769	2864	115	0.52	0.06	23688	2479	3705	287	1.43		89291	8642
	PB5	periphyton after EDTA extr.	2	23.0	4.2		16755	1819	710	433	0.18	0.12	9484	384	2070	420	1.27		33206	552
BC4	PB5	periphyton	2	68.7	1.9	11962	29777	315	31723	227	2.01	0.14	17242	280	5000	314	4.01	0.07	52429	572
	PB5	periphyton after EDTA extr.	2	41.7	2.4		17430	560	11017	2669	0.31	0.03	9442	1579	5089	972	3.54	0.0002	28512	5881
BC3	PB1	periphyton	1	16.1		1281	2498		2492		0.6		3936		8489		7.00		3009	
	PB5	periphyton	2	27.5	0.8	2269	2029	30	9133	826	5.79	1.16	8888	636	3214	396	11.86	2.37	2768	234
	PB5	periphyton after EDTA extr.	2	6.7	0.2		985	5	213	34	0.46	0.06	1638	55	2730	415	5.10	0.27	1219	245
BC2	PB1	periphyton	5	1452	66	128118	1996		2962		1.20		4326		1727				3066	
	PB2	periphyton	1	1918		238854	2412		4931		1.67		3709		2107		11.40		3864	
	PB3	periphyton	1	1907		211107	1984		925		6.71		3920		1325		9.69		3759	
	PB4	periphyton	3	2647	1263	188425	2365	452	4154	1674	1.16	0.28	4174	492	2526	997	12.04	1.01	3741	1022
TBC2-1	PB4	periphyton	3	3622	843	243086	3479	1011	4495	1572	1.38	0.23	4506	342	2291	225	11.13	0.59	4764	1069
TBC2-2	PB4	periphyton	3	3505	598	245138	2054	423	4588	316	1.36	0.07	3848	215	3229	367	10.21	1.83	2732	727
HU	PB4	periphyton (glass slide)	3	27.5	3.7	2035	4348	915	1060	117	13.01	0.88	6017	468	2985	226	6.87	0.27	6847	115
	PB4	periphyton after EDTA extr.	3	18.1	2.5	4731	3406	623	385	105	6.20	3.19	3985	245	2243	444	6.27	0.86	6978	1700
	PB5	periphyton	1	55.3			6677		2492		4.45		6489		2194		9.72		9248	
UU12	PB5	periphyton	2	120.1	7.8	1530	4241	46	4504	92	2.00	0.60	9559	800	3052	144	9.46	2.40	7339	50
	PB5	periphyton after EDTA extr.	2	77.0	32.8		3021	295	2422	1994	1.74	0.51	7454	597	2753	139	9.58	2.40	6099	490

There was no periphyton growing in UUH and RH sampling sites. *n* = number of samples. All periphyton samples from Huatarata (HU) and some of the samples from Cohana bay (BC2, BC3 and BC4)) were enriched in Ca. This enrichment is likely due to the presence of Characeae and other Ca-rich organisms or shells. Si was not analyzed by ICP-MS, but µXRF showed that this species was present as well. It may arise from the presence of diatoms and of detritic particles.

Table S6. Comparison of As bioaccumulation factors (BAFs) and As speciation for various photosynthetic organisms and natural assemblages in freshwater aquatic media

Type	Species	Sampling site or type of experiment	Environmental context or exp. conditions	Physico-chemical information on water	As speciation in water	As conc. in water (µg L⁻¹)	+/-	As conc. in biomass (mg kg⁻¹ DW)	+/-	BAF	Major As species in organism, by order of importance	Proposed mechanisms	Ref
**Microalgae or cyanobacteria**													
Green microalgae	*Chlorella vulgaris*	Lab experiment	7 days exposure	10% Bold basal growth medium, low P	As(V) (initial)	10		19.2	1.1	1920	As(V), As(III) (DMA and MMA excreted)	Reduction to As(III) and efflux (70%), formation of MMA and DMA (<10%)	Baker et al., 2016
Green microalgae	*Chlamidomonas reinhardtii*	Lab experiment	4 days exposure	WC growth medium	As(III) (initial)	997.5		6000		**6015**	As(III), As(V)	Oxydation to As(V) mainly on the cell surface, release of As(V)	Wang et al., 2014
Cyanobacteria	*Synechocystis*	Lab experiment	14 days incub.	BG-11 growth medium	As(III) (initial)	750		150		200	As(V), As(III)	Oxydation, accumulation mainly as As(V), efflux of As(III) and As(V)	Yin et al., 2012
Cyanobacteria	*Synechocystis*	Lab experiment	14 days incub.	BG-11 growth medium	As(V) (initial)	750		150		200	As(V), As(III)	Accumulation mainly as As(V), reduction to As(III), efflux of As(III) and As(V)	Yin et al., 2012
**Freshwater natural assemblages of microalgae and bacteria**													
Phytoplankton		Kam lake, Canada	Mining activities	pH 7.6 ± 0.3		145	66	894	157	6166	*		Caumette et al., 2011
Phytoplankton		Long lake, Canada	Natural enrichment	pH 7.9 ± 0.6		51	3	94	6	1843	*		Caumette et al., 2011
Phytoplankton		Grace lake, Canada	Natural enrichment	pH 7.6 ± 0.5		7	2	92	8	13143	*		Caumette et al., 2011
Small plankton (45–202 µm)		20 lakes, Northern USA	No contamination			0.04-0.59		0.13 to 10		**27902**	n.d.		Chen et a., 2000
Periphyton		Kolubara river, Serbia	Mining, industry			32		46-57		1438 - 1781	n.d.		Drndarki et al., 1993
Natural periphyton assemblages from SWRC		Lab experiment	8 days exposure			1 to 20		30-60		**3200-9700**	n.d.	Association with Fe oxides ?	Lopez et al., 2016
Biofilm		Wetlands in Idaho (USA)	Mining activities	pH 7.0 to 9.3	64% As(V), 36% As(III)	27.90	3.3	323	56	**11577**	As(III)-sulfides	Microbiological reduction of As(V) to As(III) in sulfidic conditions	Dovck et al., 2016
Microbial mats		Carnoules, France	Acid mine drainage	pH 2.5-4; 1111 mg L-1 Fe(II), precipitation zone of the AMD (oxic cond.)	> 95% As(III)	154500		131739		853	As(III)/As(V)-Fe(III) oxy-hydroxides, tooeleite	Oxidation of Fe(II) => coprecipitation of As with Fe(III) oxyhydroxides in bacterial mats.	Morin et al., 2003

Assemblage of alga and bacteria		Taupo Volcanic Zone, New Zealand	Volcanic context			32000		3019		94	n.d.		Robinson et al., 2006
Cyanobacterial biomass		growing in discharge area of tube well, Eastern India	Natural enrichment			244	4	277	22	1134	n.d.		Bhattacharya et al., 2011
**Green macroalgae**													
	Cladophora sp.	Salado river, Chile	Mining activities	pH 7.27, DO 10.2 mg L^{-1} (oxic cond.)		798		11100	300	**13910**	As(V)		Pell et al., 2013
	Cladophora sp.	San Salvador river, Chile	Mining activities	pH 8.27, DO 6.3 mg L^{-1} (oxic cond.)		1200		182	7	152	*		Pell et al., 2013
	Cladophora sp.	Danube river, Hungary	No contamination			1.1	0.2	9.33		8482	*		Schaeffer et al., 2006
	Cladophora glomerata Pilg.	Hayakawa river, Japan	Hot springs			17		18		1059	*		Miyashita et al., 2009
	Chara sp.	Loa river, Chile	Mining activities	pH 8.05, DO 8.7 mg L^{-1} (oxic cond.)		710		341	6	480	As(V), As(III)		Pell et al., 2013
**Aquatic plants**													
	Zannichellia palustris L.	Loa river, Chile	Mining activities	idem		220		79	5	359	As(V), As(III)		Pell et al., 2013
	Azolla sp.	Loa river, Chile	Mining activities	idem		220		199	12	905			Pell et al., 2013
	Potamogeton pectinatus L.	Loa river, Chile	Mining activities	idem		897		134	1	149	As(V), As(III)		Pell et al., 2013
	Potamogeton pectinatus L.	Loa river, Chile	Mining activities	idem		1400		248	2	177	As(V), As(III)		Pell et al., 2013
	Myriophyllum aquaticum L.	San Pedro river, Chile	Mining activities	pH 7.41, DO 12.6 mg L^{-1} (oxic cond.)		456		209	11	458	As(V), As(III)		Pell et al., 2013
	Callitriche stagnalis	Taupo Volcanic Zone, New Zealand	Volcanic context			90		4215		**46833**	n.d.	Sorption of As on hydrated Fe oxides on plant surface	Robinson et al., 2006
	Myriophyllum sp.	Danube river, Hungary	No contamination			1.1	0.2	5.42		**4927**	*		Schaeffer et al., 2006
**Terrestrial plant**													
	Pteris vittata (As hyperaccumulating fern)	Lab experiment	18 days expos. in hydroponics		As(V) (initial)	6225		10000		1606	>85% As(III), As(V)	Uptake of As(V), reduction to As(III), sequestration as free As(III) in vacuoles	Wang et al., 2002 Sarret et al., 2013

Table S6, cont.

	Sampling site	Campaign	Physico-chemical information on water	As speciation in water	As conc. in water (µg L^{-1})	+/-	As conc. in biomass (mg kg^{-1} DW)	+/-	BAF	Major As species in organism, by order of importance	Proposed mechanisms	Ref
**Periphyton from this study**												
Natural periphyton	lake Titicaca, BC5	PB5	pH 6.7, Eh -35 mV	72% As(V)	9.0	0.3	58.3	6.9	6459	70-90% As(V), 10-30% As(III)	As(V) intra + extracellular, As(III) intracellular	this study
Natural periphyton	lake Titicaca, BC4	PB5	pH 7.3, Eh 21 mV	68% As(V)	5.7	0.2	68.7	1.9	11962	65-85% As(V), 15-35% As(III)	idem	this study
Natural periphyton	lake Titicaca, BC3	PB1	pH 7.0, Eh 88 mV		12.6	0.3	16.1		1281			this study
Natural periphyton	lake Titicaca, BC2	PB1	pH 8.2, Eh 123 mV		11.3	0.5	1452	66	**128118**	100% As(V)	Hyperaccumulation as As(V) only	this study
Natural periphyton	lake Titicaca, BC2	PB2	pH 7.4-7.5, Eh -400 to 8.7 mV		8.0	0.1	1918		**238854**	100% As(V)	Hyperaccumulation as As(V) only	this study
Natural periphyton	lake Titicaca, BC2	PB3	pH 8.3, Eh -108 mV		9.0	0.1	1907		**211107**	100% As(V)	Hyperaccumulation as As(V) only	this study
Natural periphyton	lake Titicaca, BC2	PB4	pH 7.5		14.1	0.2	2647	1263	**188425**	100% As(V)	Hyperaccumulation as As(V) only	this study
Natural periphyton	lake Titicaca, BC3	PB5	pH 7.8, Eh -170 mV	58% As(V)	12.1	0.1	27.5	0.8	2269	45-55% A(V), 35-55% As(III)		this study
Natural periphyton	lake Titicaca, TBC2-1	PB4	pH 7.8		14.9		3622	843	**243086**	100% As(V)	Hyperaccumulation as As(V) only	this study
Natural periphyton	lake Titicaca, TBC2-2	PB4	pH 7.8		14.3		3505	598	**245138**	100% As(V)	Hyperaccumulation as As(V) only	this study
Natural periphyton	lake Titicaca, HU	PB4	pH 7.5		13.5	0.1	27.5	3.7	2035	70-75% A(V), 25-30% As(III)		this study
Natural periphyton	lake Titicaca, HU	PB5		87% As(V)	11.7	0.6	55.3		4731	42-67% As(V), 33-58% As(III)	Accumulation and/or sorption of As(V), reduction of a fraction of As(V)	this study
Natural periphyton	lake Uru Uru, UU12	PB5	pH 7.5, Eh -12 mV	85% As(V)	78.5	5.5	120.1	7.8	1530	55-70 % As(V), 30-45% As(III)	idem	this study

Bioaccumulation factor (BAF): As content in µg kg^{-1} divided by As concentration in filtered water, in µg L-1. Highest BAFs, reported in figure 2c, are highlighted in bold. * : For studies implying extraction prior to analyses, results are given only if extraction efficiency is > 80%.

Table S7: Results of the linear combination fits for the XANES spectra for the periphyton samples.

			As species (%)						As species (µg g-1 DW)[a]			
**Sample**	**Cam-paign**	**As, µg g-1 DW**	As(III)	As(V)	Arseno-sugars	MMA	Sum	R factor [b]	As(III)	As(V)	Arseno-sugars	MMA
BC5	PB5	53.4	0.26	0.74			1.00	0.0016	13.9	39.5	0.0	0
BC5-EDTA	PB5	20	0.29	0.79			1.08	0.003	5.4	14.6	0.0	0
BC4#b	PB5	67.3	0.16	0.48	0.31		0.95	0.0055	11.3	34.0	22.0	0
BC4#b-EDTA	PB5	43.4	0.28	0.38	0.32		0.98	0.0045	12.4	16.8	14.2	0
BC4#a	PB5	67.3	0.21	0.45	0.30		0.96	0.0048	14.7	31.5	21.0	0
BC4#a-EDTA	PB5	40.1	0.26	0.41	0.31		0.98	0.005	10.6	16.8	12.7	0
BC3#b	PB5	28.1	0.40	0.21	0.43		1.04	0.0015	10.8	5.7	11.6	0
BC3#a	PB5	28.1	0.21	0.32	0.43		0.96	0.0012	6.1	9.4	12.6	0
TBC2#b	PB4	3505.0		0.59		0.44	1.03	0.0009	0	2004.8	0	1500.2
TBC2#a	PB4	3622.0		0.64		0.39	1.03	0.0035	0	2250.6	0	1371.4
BC2 #b	PB4	2647.0		0.82		0.18	1.00	0.0053	0	2170.5	0	476.5
BC2 #a	PB4	2647.0	0.07	0.44		0.54	1.05	0.0026	176.5	1109.2	0	1361.3
BC2 (*FH*)	PB2	1918.0		0.63		0.41	1.04	0.0023	0	1161.9	0	756.1
BC2	PB2	1918.0		0.85		0.11	0.96	0.0031	0	1698.2	0.0	219.8
BC2	PB1	1452.0		0.55		0.46	1.01	0.0055	0	796.4	0.0	655.6
HU #b	PB5	55.3	0.14	0.70	0.12		0.96	0.0060	8.1	40.3	6.9	0
HU #a	PB5	55.3	0.35	0.37		0.28	1.00	0.0043	19.4	20.5	0.0	15.5
HU #b	PB4	27.5		0.71	0.29		1.00	0.0011	0	19.4	8.1	0
HU #a	PB4	27.5		0.64	0.36		1.00	0.0035	0	17.6	9.9	0
UU12 #b	PB5	125.6	0.08	0.64	0.25		0.97	0.003	10.4	82.9	32.4	0
UU12 #a	PB5	114.6	0.34	0.42	0.21		0.97	0.0033	40.2	49.6	24.8	0

LCFs were done in the [E_0-10; E_0+40] eV range. Up to three components were used. [a], calculated from the percentages (normalized to 100%) and As total content. [b], Fit quality criterion provided by ATHENA (R factor = $\sum[\mu_{exp} - \mu_{fit}]^2 / \sum[\mu_{exp}]^2$. *FH*: Frozen hydrated.

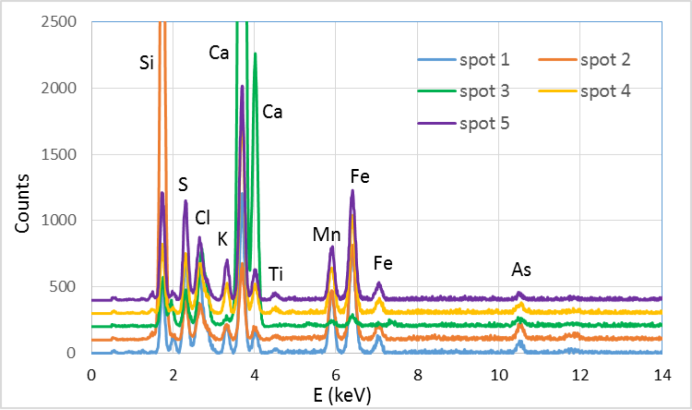

Figure S3. Examples of micro X-ray fluorescence spectra recorded on the periphyton PB4-BC2, after background subtraction. The freeze dried material was pressed into 5 mm pellets without grinding, and µXRF spectra were recorded on various spots of the pellets. Experimental conditions: 20 kV, 200 mA, 60 s acquisition time, beam diameter 300 µm. A variability in peak intensities was observed, indicating a high heterogeneity in the composition of the periphyton. As was detected in all spots, whereas it was not detected in periphyton samples from other sites.

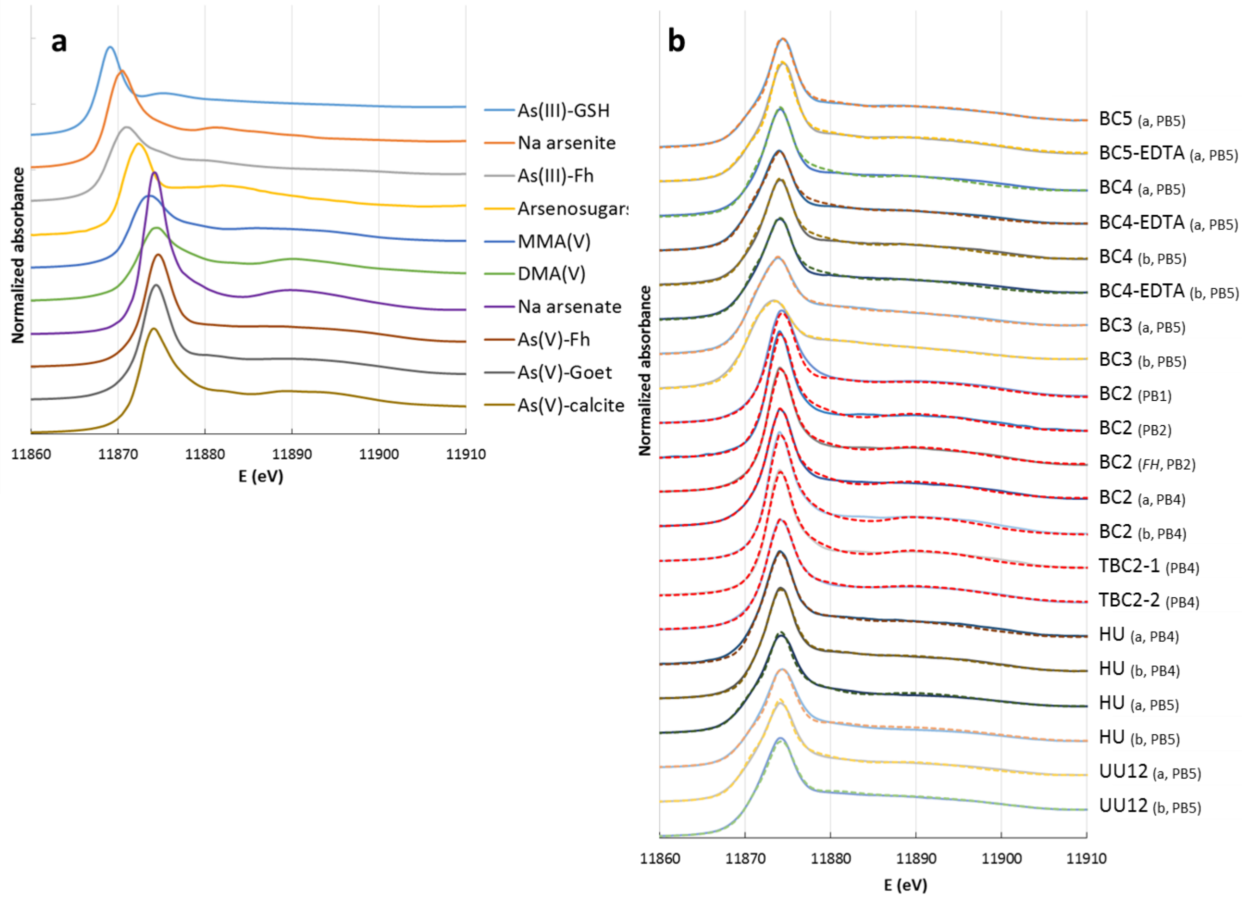

Figure S4. As K-edge XANES spectra for some As reference compounds (a) and for the periphyton samples (b, plain lines: experimental, dashed lines: linear combination fits).

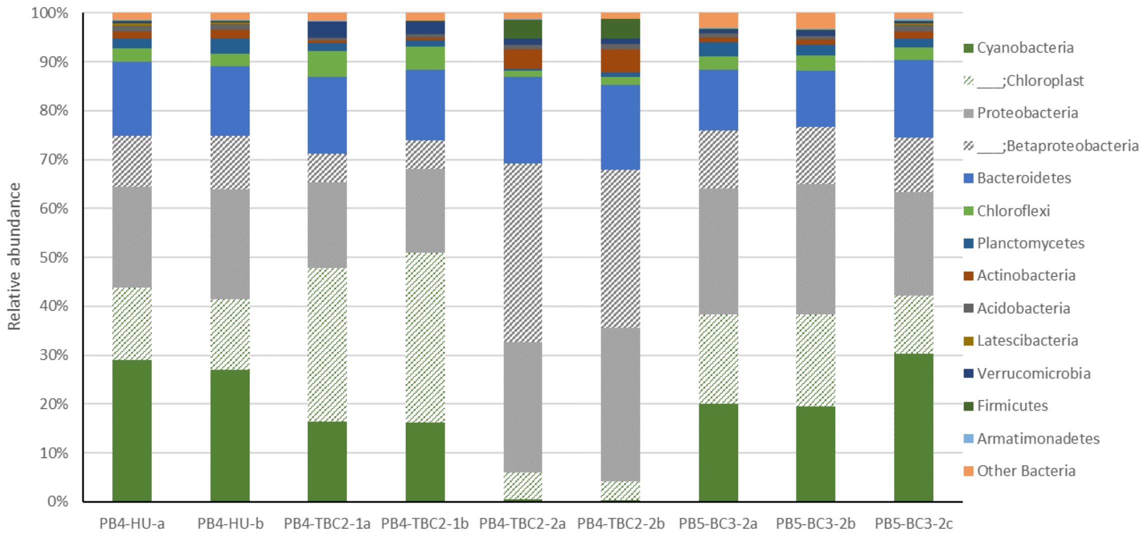

Figure S5. Structure of microbial communities of two non-hyperaccumulator periphytons (PB4-HU and PB5-BC3) and two As hyperaccumulators (PB4-TBC2-1 and PB4TBC2-2). Replicates are indicated by a lower vowel. Data represents the structure of the communities at the phylum level. Highlighted in dashed lines the class of Betaproteobacteria, belonging to Proteobacteria phylum and the Chloroplasts, related to (eucaryote) microalgae.

References

Allen, H.E., Fu, G.M. and Deng, B.L. (1993) Analysis of acid-volatile sulfdes (AVS) and simultaneously extracted metals (SEM) for the estimation of the potential toxicity in aquatic sediments. Environ. Toxicol. Chem. 12, 1441-1453.

Baker, J. and Wallschläger, D. (2016) The role of phosphorus in the metabolism of arsenate by a freshwater green alga, Chlorella vulgaris. Journal of Environmental Sciences 49, 169-178.

Bhattacharya, P. and Pal, R. (2011) Response of cyanobacteria to arsenic toxicity. Journal of Applied Phycology 23, 293-299.

Caumette, G., Koch, I., Estrada, E. and Reimer, K.J. (2011) Arsenic Speciation in Plankton Organisms from Contaminated Lakes: Transformations at the Base of the Freshwater Food Chain. Environmental Science & Technology 45, 9917-9923.

Chen, C.Y., Stemberger, R.S., Klaue, B., Blum, J.D., Pickhardt, P.C. and Folt, C.L. (2000) Accumulation of heavy metals in food web components across a gradient of lakes. Limnology and Oceanography 45, 1525-1536.

Cline, J.D. (1969) SPECTROPHOTOMETRIC DETERMINATION OF HYDROGEN SULFIDE IN NATURAL WATERS1. Limnology and Oceanography 14, 454-458.

Di Toro, D.M., McGrath, J.A., Hansen, D.J., Berry, W.J., Paquin, P.R., Mathew, R., Wu, K.B. and Santore, R.C. (2005) Predicting sediment metal toxicity using a sediment biotic ligand model: Methodology and initial application. Environ. Toxicol. Chem. 24, 2410-2427.

Dovick, M.A., Kulp, T.R., Arkle, R.S. and Pilliod, D.S. (2016) Bioaccumulation trends of arsenic and antimony in a freshwater ecosystem affected by mine drainage. Environmental Chemistry 13, 149-159.

Drndarski, N., Stojic, D. and Markov, Z. (1993) STABLE ISOTOPES IN PERIPHYTON AND SEDIMENTS FROM THE KOLUBARA RIVER AND ITS TRIBUTARIES. Environmental Pollution 80, 287-292.

Escudié, F., Auer, L., Bernard, M., Mariadassou, M., Cauquil, L., Vidal, K., Maman, S., Hernandez-Raquet, G., Combes, S. and Pascal, G. (2018) FROGS: Find, Rapidly, OTUs with Galaxy Solution. Bioinformatics 34, 1287-1294.

Fishman, L. and Friedman, L. (1989) Methods for determination of inorganic substances in water and fluvial sediments, Book 5, Chapter A1. USGS.

Guedron, S., Point, D., Acha, D., Bouchet, S., Baya, P.A., Tessier, E., Monperrus, M., Molina, C.I., Groleau, A., Chauvaud, L., Thebault, J., Amice, E., Alanoca, L., Duwig, C., Uzu, G., Lazarro, X., Bertrand, A., Bertrand, S., Barbraud, C., Delord, K., Gibon, F.M., Ibanez, C., Flores, M., Saavedra, P.F., Ezpinoza, M.E., Heredia, C., Rocha, F., Zepita, C. and Amouroux, D. (2017) Mercury contamination level and speciation inventory in Lakes Titicaca & Uru-Uru (Bolivia): Current status and future trends. Environmental Pollution 231, 262-270.

Lopez, A.R., Hesterberg, D.R., Funk, D.H. and Buchwalter, D.B. (2016) Bioaccumulation Dynamics of Arsenate at the Base of Aquatic Food Webs. Environmental Science & Technology 50, 6556-6564.

Meylan, S., Behra, R. and Sigg, L. (2004) Influence of metal speciation in natural freshwater on bioaccumulation of copper and zinc in periphyton: A microcosm study. Environmental Science & Technology 38, 3104-3111.

Miyashita, S., Shimoya, M., Kamidate, Y., Kuroiwa, T., Shikino, O., Fujiwara, S., Francesconi, K.A. and Kaise, T. (2009) Rapid determination of arsenic species in freshwater organisms from the arsenic-rich Hayakawa River in Japan using HPLC-ICP-MS. Chemosphere 75, 1065-1073.

Morin, G., Juillot, F., Casiot, C., Bruneel, O., Personne, J.C., Elbaz Poulichet, F., Leblanc, M., Ildefonse, P. and Calas, G. (2003) Bacterial formation of tooeleite and mixed Arsenic(III) or Arsenic(V)-Iron(III) gels in the carnoulbs acid mine drainage, France. A XANES, XRD, and SEM study. Environmental Science and Technology 37, 1705-1712.

Pell, A., Marquez, A., Lopez-Sanchez, J.F., Rubio, R., Barbero, M., Stegen, S., Queirolo, F. and Diaz-Palma, P. (2013) Occurrence of arsenic species in algae and freshwater plants of an extreme arid region in northern Chile, the Loa River Basin. Chemosphere 90, 556-564.

Reese, B.K., Finneran, D.W., Mills, H.J., Zhu, M.-X. and Morse, J.W. (2011) Examination and Refinement of the Determination of Aqueous Hydrogen Sulfide by the Methylene Blue Method. Aquatic Geochemistry 17, 567.

Robinson, B., Kim, N., Marchetti, M., Moni, C., Schroeter, L., van den Dijssel, C., Milne, G. and Clothier, B. (2006) Arsenic hyperaccumulation by aquatic macrophytes in the Taupo Volcanic Zone, New Zealand. Environmental and Experimental Botany 58, 206-215.

Sarret, G., Pilon Smits, E., Castillo-Michel, H., Isaure, M.P., Zhao, F.J. and Tappero, R. (2013) Use of synchrotron-based techniques to elucidate metal uptake and metabolism in plants. Adv. Agron. 119, 1-82, DOI 10.1016/B1978-1010-1012-407247-407243.400001-407249.

Schaeffer, R., Francesconi, K.A., Kienzl, N., Soeroes, C., Fodor, P., Váradi, L., Raml, R., Goessler, W. and Kuehnelt, D. (2006) Arsenic speciation in freshwater organisms from the river Danube in Hungary. Talanta 69, 856-865.

Small, J.M. and Hintelmann, H. (2007) Methylene blue derivatization then LC–MS analysis for measurement of trace levels of sulfide in aquatic samples. Analytical and Bioanalytical Chemistry 387, 2881-2886.

Small, J.M. and Hintelmann, H. (2014) Sulfide and mercury species profiles in two Ontario boreal shield lakes. Chemosphere 111, 96-102.

Wang, J., Zhao, F.-J., Meharg, A.A., Raab, A., Feldmann, J. and McGrath, S.P. (2002) Mechanisms of Arsenic Hyperaccumulation in Pteris vittata. Uptake Kinetics, Interactions with Phosphate, and Arsenic Speciation. Plant Physiology 130, 1552-1561.

Wang, N.X., Huang, B., Xu, S., Wei, Z.B., Miao, A.J., Ji, R. and Yang, L.Y. (2014) Effects of nitrogen and phosphorus on arsenite accumulation, oxidation, and toxicity in Chlamydomonas reinhardtii. Aquatic Toxicology 157, 167-174.

Wang, Y. and Qian, P.-Y. (2009) Conservative Fragments in Bacterial 16S rRNA Genes and Primer Design for 16S Ribosomal DNA Amplicons in Metagenomic Studies. PLOS ONE 4, e7401.

Yin, X.X., Wang, L.H., Bai, R., Huang, H. and Sun, G.X. (2012) Accumulation and Transformation of Arsenic in the Blue-Green Alga Synechocysis sp PCC6803. Water Air and Soil Pollution 223, 1183-1190.
